# The IRE1‐XBP1s Axis Drives Inflammatory Osteolysis by Regulating a 5‐HT Dependent Endogenous Anti‐Autophagy Mechanism

**DOI:** 10.1002/advs.76755

**Published:** 2026-07-23

**Authors:** Pengchao Yang, Binxiang Zhu, Yuzhi He, Yang Tian, Honglei Kang, Shian Hu, Pengju Wang, Yong Xu, Zhuowei Lei, Peijun Qi, Hao Yang, Yang Lin, Yimin Dong, Feng Li, Hanfeng Guan

**Affiliations:** ^1^ Department of Orthopedics Tongji Hospital Tongji Medical College Huazhong University of Science and Technology Wuhan China; ^2^ Hubei Provincial Clinical Medical Research Center for Spinal Surgery Wuhan Hubei China; ^3^ Department of Spinal Surgery Ningbo No.2 Hospital Ningbo China; ^4^ Department of Spinal Surgery Ezhou Central Hospital Ezhou China

**Keywords:** 3MA, 5‐HT, autophagy, inflammatory osteolysis, osteoclast

## Abstract

Inflammatory osteolysis arises from pro‐inflammatory cytokine‐driven osteoclast activation and disrupted bone remodeling equilibrium. The IRE1‐XBP1s axis, a major unfolded protein response pathway, regulates cellular homeostasis, but its role in inflammatory osteoclastogenesis remained unexplored. Single‐cell RNA‐seq showed increased osteoclast precursor cells and activated IRE1‐XBP1s in LPS‐induced osteolysis. Inhibition of this axis reduced osteoclastogenesis and bone loss in vitro and in vivo. RNA‐seq indicated that blocking IRE1‐XBP1s suppressed Slc6a4 transcription, with gene set enrichment analysis confirming its role in 5‐HT transport. Dual‐luciferase assays and ChIP‐PCR demonstrated XBP1s’ direct transcriptional regulation targeting the Slc6a4 promoter. The 5‐HT transporter inhibitor escitalopram also inhibited osteoclastogenesis, highlighting the IRE1‐XBP1s‐Slc6a4 axis's importance. Notably, untargeted metabolomics suggested 5‐HT inhibited intracellular 3‐methyladenine (3MA) metabolism, a compound previously considered unnatural. HPLC‐MS confirmed the presence of 3MA metabolism in inflammatory osteoclasts, and 3MA supplementation attenuated 5‐HT‐induced autophagy and osteoclast differentiation. Blocking the IRE1‐XBP1s‐Slc6a4 axis reduced pro‐osteoclastogenic effects in inflammatory bone disease patient‐derived PBMCs. This study demonstrates that IRE1‐XBP1s inhibition alleviates inflammatory osteoclastogenesis and osteolysis via a 5‐HT‐dependent anti‐autophagy mechanism, proposing this pathway as a therapeutic target for inflammatory bone loss.

## Graphical abstract

A previously unrecognized IRE1‐XBP1s‐Slc6a4 signaling axis links endoplasmic reticulum stress to serotonin metabolism, autophagy, and inflammatory osteoclastogenesis. By promoting intracellular serotonin uptake and reducing endogenous 3‐methyladenine accumulation, this pathway accelerates inflammatory bone destruction and provides a promising therapeutic target for osteolytic diseases.

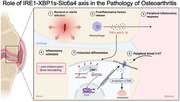

**Abbreviations**
3MA: 3‐methyladenine
5‐HT: 5‐hydroxytryptamine
5‐HTT: 5‐HT transporter
BMDM: bone marrow derived macrophage
CMP: common myeloid progenitor
DEGs: differential expression genes
ER: endoplasmic reticulum
HD: heathy donors
IRE1: inositol‐requiring enzyme 1
KEGG: Kyoto Encyclopedia of Genes and Genomes
LPS: lipopolysaccharide
M‐CSF: macrophage colony‐stimulating factor
MDP: monocyte‐dendritic cell progenitors
MS: mass spectrometry
OA: osteoarthritis
PAR: resorption pit area ratio‌
PBMC: peripheral blood mononuclear cells
PCA: principal component analysis
RA: rheumatoid arthritis
RANKL: receptor activator of nuclear factor κB ligand
RPC: resorption pit count‌
RT: retention time
TG: Thapsigargin
TNF‐α: tumor necrosis factor α
TRAP: tartrate‐resistant acid phosphatase
XBP1s: X box‐binding protein‐1
UPR: unfolded protein response

## 1 Introduction

Inflammatory osteolysis is a common pathological feature in diseases such as rheumatoid arthritis (RA), osteomyelitis, and periprosthetic osteolysis [1, 2, 3]. The pathological hallmark of inflammatory osteolysis is the aberrant activation of osteoclasts in response to pro‐inflammatory factors such as tumor necrosis factor α (TNF‐α) [4, 5]. As a dynamically remodeling tissue, bone homeostasis is exquisitely regulated by osteoclast‐mediated bone resorption and osteoblast‐mediated bone formation [6, 7]. The excessive production of inflammatory cytokines at the lesion site disrupts this equilibrium and subsequently leads to bone loss. Exemplified by RA, it is the most prevalent chronic systemic disabling disease among autoimmune disorders and is characterized by progressive joint destruction and bone erosion [1, 8]. However, the molecular mechanisms underlying inflammatory bone resorption in RA remain equivocal, and there is no drug specifically targeting inflammatory osteoclasts for RA [9, 10, 11]. Exploring novel therapeutic targets for inflammatory osteolysis is essential to reduce the disease burden and improve patients' quality of life.

The unfolded protein response (UPR) is a homeostatic regulatory mechanism activated during endoplasmic reticulum (ER) stress, whose intensity and duration critically determine cell fate and are closely linked to the pathogenesis of diverse diseases [12, 13, 14]. Inositol‐requiring enzyme 1 (IRE1), an ER‐resident sensor, detects the accumulation of misfolded proteins within the ER lumen and initiates the UPR [12, 15]. Through its RNase domain, IRE1 catalyzes the unconventional splicing of XBP1u mRNA to produce XBP1s mRNA, which encodes a potent transcription factor [12]. This factor translocates to the nucleus to modulate the expression of genes involved in protein folding, degradation, and ER homeostasis, thereby orchestrating adaptive or apoptotic outcomes under stress conditions. Recent studies have indicated that the IRE1‐XBP1s pathway can influence bone homeostasis by modulating the receptor activator of nuclear factor κB ligand (RANKL) mediated downstream molecular mechanisms in murine ovariectomy models [12, 16, 17]. However, the role of the IRE1‐XBP1s pathway in inflammatory osteolysis models remains to be further elucidated [18].

Beyond its canonical role in maintaining ER proteostasis, emerging evidence suggests that the IRE1‐XBP1s pathway also participates in metabolic adaptation by reshaping transcriptional programs involved in metabolite transport. Among these metabolic mediators, 5‐hydroxytryptamine (5‐HT) has attracted increasing attention due to its pleiotropic functions in immunity and bone remodeling. Synthesized by enterochromaffin cells, 5‐HT (also known as serotonin) not only functions as a neurotransmitter but also plays pivotal roles in inflammation, immunity, and tumorigenesis [19, 20, 21, 22]. Previous studies have demonstrated that 5‐HT is closely associated with bone homeostasis, and its metabolic dysregulation contributes to skeletal disorders [23]. For instance, centrally derived 5‐HT promotes bone formation by activating 5‐HT_1_A receptors on sympathetic nerves and relieving their inhibitory effect on osteoblasts. In contrast, peripherally derived 5‐HT directly binds to 5‐HT_2_B receptors on osteoblasts, exerting an inhibitory effect on bone formation [23, 24, 25]. In addition, 5‐HT also regulates osteoclast function. During systemic inflammatory responses, the release of 5‐HT from platelets‌ elevates its levels in peripheral tissues [26, 27]. ‌5‐HT can modulate macrophage activity to promote the release of inflammatory cytokines such as IL‐6 and TNF‐α, while simultaneously activating the NF‐κB pathway [28]. Through the ‌non‐classical NF‐κB pathway‌, it disrupts vascular endothelial integrity and increases microvascular exudation, which are typical pathological features of inflammation. However, whether peripheral 5‐HT mediates the initiation and progression of inflammatory osteolysis remains unknown.

Because metabolic remodeling and autophagic adaptation are tightly coupled during osteoclast differentiation and inflammatory activation, we next explored whether the IRE1‐XBP1s regulated 5‐HT axis contributes to downstream metabolic‐autophagic reprogramming. In the present study, we found that targeting the IRE1‐XBP1s pathway potently suppressed both human and mouse inflammatory osteoclastogenesis and attenuated inflammatory osteolysis. Using multi‐omics, we revealed that the IRE1‐XBP1s pathway regulates cellular 5‐HT transport via transcriptionally activating *Slc6a4*, which encodes the serotonin transporter in osteoclasts. In addition, we found that 5‐HT promotes inflammatory osteoclast differentiation via decreasing the endogenous levels of 3‐methyladenine (3MA). Conventionally, 3MA is thought to be an artificially synthesized autophagy inhibitor. However, we found that 3MA is also naturally present in osteoclasts, constituting an endogenous anti‐autophagy system, which is under the regulation of the IRE1‐XBP1s‐5‐HT pathway. Collectively, our findings uncover a previously unrecognized IRE1‐XBP1s‐Slc6a4‐3MA signaling axis that integrates ER stress adaptation, metabolic remodeling, and endogenous autophagy regulation during inflammatory osteolysis. Targeting this signaling axis may represent a promising therapeutic strategy for inflammatory bone loss diseases.

## 2 Methods

The key resources are as described in Table 1.

**TABLE 1:** It summarizes the sources and identifiers of all the reagents used in this work.

Reagents or Resource	Source	Identifier
Antibodies		
Anti‐ IRE1 antibody	ABclonal	Cat# A21021
Anti‐ p‐IRE1 antibody	ABclonal	Cat# AP1442
Anti‐ β‐actin antibody	ABclonal	Cat# AC004
Anti‐ LC3A/LC3B	ABclonal	Cat# A27200PM
Anti‐ Beclin1 antibody	ABclonal	Cat# A21191
Anti‐ Mmp9 antibody	Proteintech	Cat#10375‐2‐AP
Anti‐ Slc6a4 antibody	Proteintech	Cat#19559‐1‐AP
Anti‐ Trap antibody	Abcam	Cat# ab96372
Anti‐ Ctsk antibody (for WB)	Abcam	Cat# ab207086
Anti‐ Ctsk antibody (for IF)	Santa Cruz	Cat# sc‐48353
Anti‐ XBP1s antibody (for WB&ChIP)	Cell Signaling Technology	Cat# E9V3E
Chemicals, peptides and recombinant proteins		
Recombinant human M‐CSF	PEPROTECH	Cat# 300–25
Recombinant human RANK ligand	PEPROTECH	Cat# 310‐01
Recombinant mouse M‐CSF	R&D Systems	Cat# 416‐ML
Recombinant mouse RANK ligand	R&D Systems	Cat# 462‐TR
4μ8C	Selleck	Cat# S7272
Lipopolysaccharide	Sigma‐Aldrich	Cat# L2630
FITC‐phalloidin	Sigma‐Aldrich	Cat# P5282
Escitalopram	MedChemExpress	Cat# HY‐14258
3‐methyladenine	MedChemExpress	Cat# HY‐19312
5‐HT	MedChemExpress	Cat#HY‐B1473A
Asenapine	MedChemExpress	Cat# HY‐10121
Critical commercial assays		
Mouse 5‐HT ELISA kit	BBI life sciences corporation	Cat# D751013
ChIP assay kit	Beyotime	Cat# P2078
TRAP staining kit	Sigma‐Aldrich	NA

### 2.1 Single‐Cell RNA‐Sequencing Data Processing and Analysis

The bone marrow cells were diluted and collected from bilateral femurs of sham and LPS‐challenged mice to generate single‐cell gene expression libraries. After thoroughly dissociating the bone marrow cells, the cell suspensions were filtered through 40‐µm Flowmi cell strainer to remove cell debris. Then, the suspension was centrifuged at 300×g for 6 min to remove the supernatant. Red blood cells were removed with red blood cell lysis buffer, and the count and viability of the remaining cells were estimated using a fluorescence Cell Analyzer (Countstar Rigel S2) with acridine orange/propidium iodide reagent. Finally, the single‐cell RNA‐sequencing libraries were prepared using a SeekOne MM Single Cell 3' library preparation kit (SeekGene Catalog No. K00104) and then sequenced on an Illumina NovaSeq 6000 with PE150 read length.

The raw single‐cell RNA‐sequencing data from different samples were integrated using the Seurat package, and batch effects were adjusted using the Harmony package in R software. For data analysis, the Seurat package was used to perform nonlinear dimensional reduction. A total of 2500 highly variable genes were selected for principal component analysis (PCA). The functions RunUMAP and FindClusters were used for plotting. The FindAllMarkers function was used to identify differentially expressed genes (DEGs), and the ggplot2 package was used for plotting. Osteoclasts in the bone marrow differentiate from the myeloid lineage of bone marrow cells that include common myeloid progenitor (CMP), monocyte‐dendritic progenitor (MDP), dendritic cells, monocytes, and macrophages. We defined c‐Fms^+^ monocytes, macrophages, and dendritic cells in the bone marrow as osteoclast precursors that possess the ability to differentiate into mature osteoclasts.

### 2.2 Human Osteoclast Differentiation In Vitro

Venous blood (10 mL) was obtained from healthy donators and patients clinically diagnosed with osteomyelitis, RA, or OA.‌ Blood samples were carefully layered onto the surface of FICOLL (Solarbio, Cat# P8900) separation medium along the tube wall. The sample was then centrifuged using a ‌swinging‐bucket rotor‌ to separate the blood components into distinct layers at a centrifugal force of 800×g and an acceleration/deceleration rate of 2. The thin, intermediate layer containing peripheral blood mononuclear cells (PBMCs) was gently aspirated using a Pasteur pipette and transferred to a new centrifuge tube. The cells were washed and resuspended in PBS buffer, followed by centrifugation at 400×g with an acceleration/deceleration rate of 9. The washing step was repeated twice. Subsequently, the cells were resuspended in adherent culture medium (containing 1% fetal bovine serum (FBS), 1% GlutaMAX, and 1% penicillin/streptomycin) at a density of 3 × 10^6^ cells/mL and seeded into a 10 cm culture dish. The dish was then incubated for 1.5 h at 37°C with 5% CO_2_ to purify the mononuclear cells. Non‐adherent cells were discarded together with the supernatant.

Adherent cells were cultured in osteoclast differentiation medium, containing 10% FBS, 1% GlutaMAX, 1% penicillin/streptomycin, and 30 ng/mL Human M‐CSF (PEPROTECH, Cat# 300–25), for 7 to 10 days to induce macrophages. On day 3 and day 5, 2 mL and 4 mL of fresh osteoclast medium were supplemented, respectively. Although the cells ultimately used for the RNA‐seq and osteoclast differentiation assays were essentially macrophages derived from PBMCs, they were still described as PBMCs to distinct from murine macrophages [29]. After trypsin digestion, cells were seeded in a 12‐well plate at a density of 3 × 10^5^ cells per well and were cultured overnight. Osteoclast differentiation was induced with 30 ng/mL Human RANKL (PEPROTECH, Cat# 310‐01), with medium replaced every 2 days until mature osteoclasts were observed. The experiments were ethically approved by the Ethics Committee of Tongji Hospital, Tongji Medical College, Huazhong University of Science and Technology (Approval No. TJ‐IRB202508030). Written informed consent was obtained from all participants prior to blood collection in accordance with the Declaration of Helsinki.

### 2.3 Bone Marrow Derived Macrophages (BMDMs) Isolation and LPS‐Mediated Osteoclast Differentiation In Vitro

Osteoclast differentiation was induced from BMDMs obtained from the bone marrow of bilateral femurs and tibias of 8‐week‐old male wide type C57BL/6J mice. Bone marrow cells were diluted and cultured in alpha‐modified Eagle's medium (α‐MEM; BOSTER Bio; Wuhan, China) containing 30 ng/mL mouse M‐CSF (R&D Systems, Cat# 416‐ML), 10% fetal bovine serum, and 1% penicillin/streptomycin in 10‐cm dishes for 16 h. The supernatants were then transferred to a new 10 cm dish and cultured for 48h in 37°C incubator. BMDMs adhering to the bottom were used for subsequent experiments. To induce inflammatory osteoclast differentiation, BMDMs were cultured in 96‐well plates at a density of 2 × 10^4^ cells per well. On the first day, BMDMs were pre‐stimulated with α‐MEM medium containing 30 ng/mL M‐CSF and 100 ng/mL mouse RANKL (R&D Systems, Cat# 462‐TR) for 24 h, followed by induction with inflammatory medium (as described below) containing ‌only 30 ng/mL M‐CSF, 10 ng/mL RANKL, and 80 ng/ml LPS for 3 days with daily medium replacement, until significant mature osteoclast formation was observed.

### 2.4 Osteoclasts TRAP Staining and Actin Ring Staining

Mature inflammatory osteoclasts were washed with PBS and fixed in 4% paraformaldehyde for 15 min. The tartrate‐resistant acid phosphatase (TRAP) staining was performed with the TRAP staining kit (Sigma–Aldrich). The staining buffer was prepared according to the manufacturer's instructions and was added to the cells, followed by incubation at 37°C for 1 h. After staining, multinucleated cells were counted as osteoclasts with at least three nuclei, and representative images were taken under the microscope. For actin ring staining, mature osteoclasts were permeabilized with 0.25% Triton X‐100 for 15 min and were incubated with FITC‐phalloidin (Sigma–Aldrich, Cat# P5282) at room temperature for 1 h. The actin ring structures, including podosome clusters and podosome belts, were counted under a fluorescence microscope, and representative images were captured for each well.

### 2.5 Cell Toxicity Assay

BMDMs were seeded onto 96‐well plates at a density of 1 × 10^4^ per well and were cultured with 30 ng/mL M‐CSF for 24 h. Then, the medium was replaced with fresh medium containing various concentrations of 4μ8C (Selleck, Cat# S7272), escitalopram (ESC; MedChemExpress, Cat# HY‐14258), asenapine (MedChemExpress, Cat# HY‐10121) or 3‐methyladenine (3MA; MedChemExpress, Cat# HY‐19312) once daily for two days. CCK8 assay kit (Cat# AR1160, Boster Biotechnology, Wuhan, China) was used to examine the influence of escitalopram, asenapine, and 3MA on cell viability of BMDMs.

### 2.6 RNA‐Sequencing and Bioinformatic Analysis

Cells used for RNA‐seq were cultured in 6‐well plates at a density of 1 × 10^6^ per well. After intervention for two days, the cells were lysed with 1 mL Trizol (Takara, Japan) to extract the RNA. The samples were sent to Novogene Co., Ltd (Beijing, China) for further processing and sequencing. RNA‐sequencing data were analyzed using R software (version 4.1.3). We identified DEGs by setting the threshold of |log2 Fold change| as greater than 0.5 and the P value less than 0.05. Kyoto Encyclopedia of Genes and Genomes (KEGG) analysis was then performed to determine significantly enriched pathways of the DEGs. In addition, we also performed GSEA for two‐group sequencing to investigate the changes in the pathways of interest. The RNA‐sequencing data that support the findings of this study have been deposited in GEO.

### 2.7 Untargeted Metabolomics Detection and Analysis

For untargeted metabolomics detection, 5 × 10^6^ BMDMs cells on day 1 were cultured in osteoclast pre‐stimulation medium with 30 ng/mL M‐CSF and 100 ng/mL RANKL in a 10 cm dish for 24 h. Then, the treated group was then continuously intervened for the next two days with the inflammatory medium containing 8 µm 5‐HT. On day 4, the cells were scraped and collected in EP tubes and resuspended with prechilled 80% methanol by well vortex. Then, the samples were melted on ice and whirled for 30 s. After sonification for 6 min, cells were centrifuged at 5,000 rpm, 4°C for 1 min. The supernatant was freeze‐dried and dissolved with 10% methanol. Finally, the solution was injected into the LC‐MS system for analysis in Novogene Co., Ltd (Beijing, China). The analysis of the non‐targeted metabolomics data was performed in the MetaboAnalyst platform (V5.0): https://www.metaboanalyst.ca/. In the platform, the statistical analysis module was used for principal component analysis and fold‐change calculation. In this module, the orthogonal Partial Least Squares – Discriminant Analysis (orthoPLS‐DA) model was used to calculate variable importance in projection (VIP). We used VIP > 1, |log2 Fold change)| > 0.5, and P < 0.05 as benchmarks to screen for differential metabolites. Finally, we analyzed the involved pathway of the identified differential metabolites in the enrichment analysis module.

### 2.8 Mice and Experiments

All of the animal experimental protocols and procedures in the study were approved by the Ethics Committee of Tongji Hospital, Tongji Medical College, Huazhong University of Science and Technology. Five‐week‐old male C57BL/6J mice were purchased via the experimental animal center of our institution. The mice were further housed in a specific pathogen‐free environment, and they were fed with sterile food and water in condition of 12‐h light/dark cycle and a constant temperature around 25°C. The LPS‐mediated mouse cranial inflammatory osteolysis model was conducted when the mice became six weeks old. Briefly, after anesthesia with isoflurane, mice were injected with LPS (Sigma‐Aldrich, Cat# L2630) at the cranial suture site [5]. The initial dose was 10 mg/kg, followed by subsequent doses of 15 mg/kg administered every 5 days until sacrifice on day 15. The control mice were injected with an equivalent volume of sterile PBS solution at the cranial suture site.

For pharmacological intervention, the drugs, including 4μ8C, 5‐HT (MedChemExpress, Cat# HY‐B1473A), escitalopram, and 3MA, were dissolved in vehicles (VEH, 10% v/v dimethyl sulfoxide + 40% v/v polyethylene glycol 300 + 5% v/v Tween 80 + 45% v/v ddH_2_O) and were administrated via intraperitoneal injection. The drugs were administrated once daily for a continuous 15 days until the end of the experiment. Finally, the mice were euthanized, and the murine entire calvaries were harvested and fixed in a 4% polyformaldehyde solution for subsequent micro‐CT bone morphological analysis and histological staining. Hearts, livers, and kidneys from the experimental were also collected for hematoxylin and eosin (H&E) staining to assess potential drug‐induced organ toxicity.

### 2.9 Micro‐Computer Tomography (µCT) Analysis

After the in vivo interventions, the mice were sacrificed, and the entire skull of each experimental mouse was collected and fixed in 4% paraformaldehyde for 72 h. Then, the skulls were scanned in the µCT system (Scanco Medical) for bone morphological analysis. Scanning parameters were set at 55 kV, 230 ms and 12 µm, with a resolution of 10 per pixel. We used the built‐in software of the µCT for three‐dimensional reconstruction, and the morphological analysis was calculated using ImageJ software. Given the thinness of murine skull bones and the indistinct boundary between cortical and trabecular bone, we employed alternative morphological parameters to minimize analytical errors. Specifically, the region of interest (ROI) was defined as the diamond‐shaped area bounded by the sagittal suture (anterior‐posterior vertices) and coronal suture (left‐right vertices). Key morphological parameters included: (1) Resorption pit arearatio‌ (PAR)– Calculated as the proportion of completely eroded bone area relative to the total ROI area; (2) Resorption pitcount‌ (RPC)– Quantified as the number of cortical bone defects (incomplete erosion) within the ROI.

### 2.10 Histomorphometry Analysis

After µCT scanning, mouse skulls were decalcified with 10% EDTA decalcification solution for 3 weeks. The samples were then paraffin‐embedded and sectioned at 5 µm thickness. Osteoclast formation was evaluated by TRAP staining. The typical images of the areas of interest on the stained sections were taken at the same region of each cranial slice. The histomorphometric parameters, including osteoclast number per bone perimeter (N.OC/B.Pm) and osteoclast surface (OC.S/B.S), were analyzed in the Osteomeasure Analysis System (Osteometrics). Osteoblast formation was evaluated by toluidine blue staining. The histomorphometric parameters, including osteoblast number per bone perimeter (N.OB/B.Pm) and osteoclast surface (OB.S/B.S). All analyses were performed according to the recommendations of the Nomenclature Committee of the American Society for Bone and Mineral Research [30].

### 2.11 Immunofluorescence Staining

Immunofluorescence staining was performed on the paraffin‐embedded cranial sections after dewaxing. Before staining, bone sections were blocked with 5% bovine serum albumin solution for 30 min. Subsequently, the blocking solution was removed, and the sections were incubated with the primary antibody at 4°C overnight. The next day, the cranial sections were washed for three times, followed by incubation with the second fluorescent antibody of the same origin to the primary antibody in a darkroom for 1 h. The nuclei were stained with DAPI solution at room temperature for 10 min. All the images were obtained under a fluorescence microscopy (#80i, Nikon, Japan). The primary antibody used for immunofluorescence staining was CTSK (Santa Cruz Biotechnology, Cat#sc‐48353).

### 2.12 Mass Spectrometry Focusing on 3‐Methyladenine

For mass spectrometry (MS), a total of 6 × 10^6^ cells were cultured in 10‐cm dish. Upon harvesting, the cells were scraped and were centrifuged at 2000 rpm for 5 min, followed by washing with sterile PBS before being used for MS. For the detection of endogenous 3MA, 10 mg of 3MA standard (MedChemExpress, Cat# HY‐19312) was dissolved in acetonitrile solution and serially diluted to prepare working standard solutions at different concentration gradients for calibration curve generation; subsequently, 80 µL methanol and acetonitrile were added to each cell sample, followed by vortex mixing and cell lysis through three cycles of homogenization using a low‐temperature vortex homogenizer, after which the samples were incubated at −20°C overnight for reaction and extraction, and finally centrifuged at 4°C and 12,000 × g to collect the supernatant. Then, the establishment of chromatographic and mass spectrometric conditions comprised chromatographic parameters and MS detection settings: Chromatographic separation was achieved using an Alphasil VC‐C18AQ column (2.1 × 100 mm, 2.5 µm) maintained at 35°C, with the mobile phase consisting of (A) 0.1% formic acid in water and (B) 0.1% formic acid in acetonitrile, employing a 2 µL injection volume; for the target analyte 3 MA, mass spectrometric detection was optimized with the quantitative transition monitored at m/z 133.1 and a retention time (RT) of 0.839 min. The MS data were analyzed by Scale Biomedicine Technology Co., LTD (Beijing, China).

### 2.13 Statistical Analysis

Data from the current study were analyzed using GraphPad Prism 9. Each experiment was performed in three independent replicates. Data were expressed as mean ± SEM (standard error of mean) for each group. Unpaired Student t‐test was used to compare differences between two groups, while one‐way or two‐way ANOVA followed by Bonferroni multiple comparison test was used to compare differences among three or more groups. The sample sizes of animal studies and the replicates of cell experiments in each statistical analysis are indicated in the figure legends. In all statistical tests, a P‐value less than 0.05 was considered statistically significant.

## 3 Results

### 3.1 LPS Induces Human and Mouse Inflammatory Osteoclastogenesis

LPS is a potent inflammation driver and has been used to induce inflammatory osteoclast differentiation in mouse models [5, 31, 32, 33, 34]. We confirmed that LPS dose‐dependently promotes osteoclast differentiation from mouse BMDMs at a concentration of 20 ∼ 80 ng/mL (Figure S1A–C), accompanied by increased expression of osteoclast marker genes including *Ctsk*, *Mmp9*, and *Trap* (Figure S1D). The formation of the actin ring was also enhanced by LPS treatment (Figure S1E,F). In addition, LPS potently induced the expression of proinflammatory factors such as *IL‐1β* and *TNFα* (Figure S1G).

**FIGURE 1:**
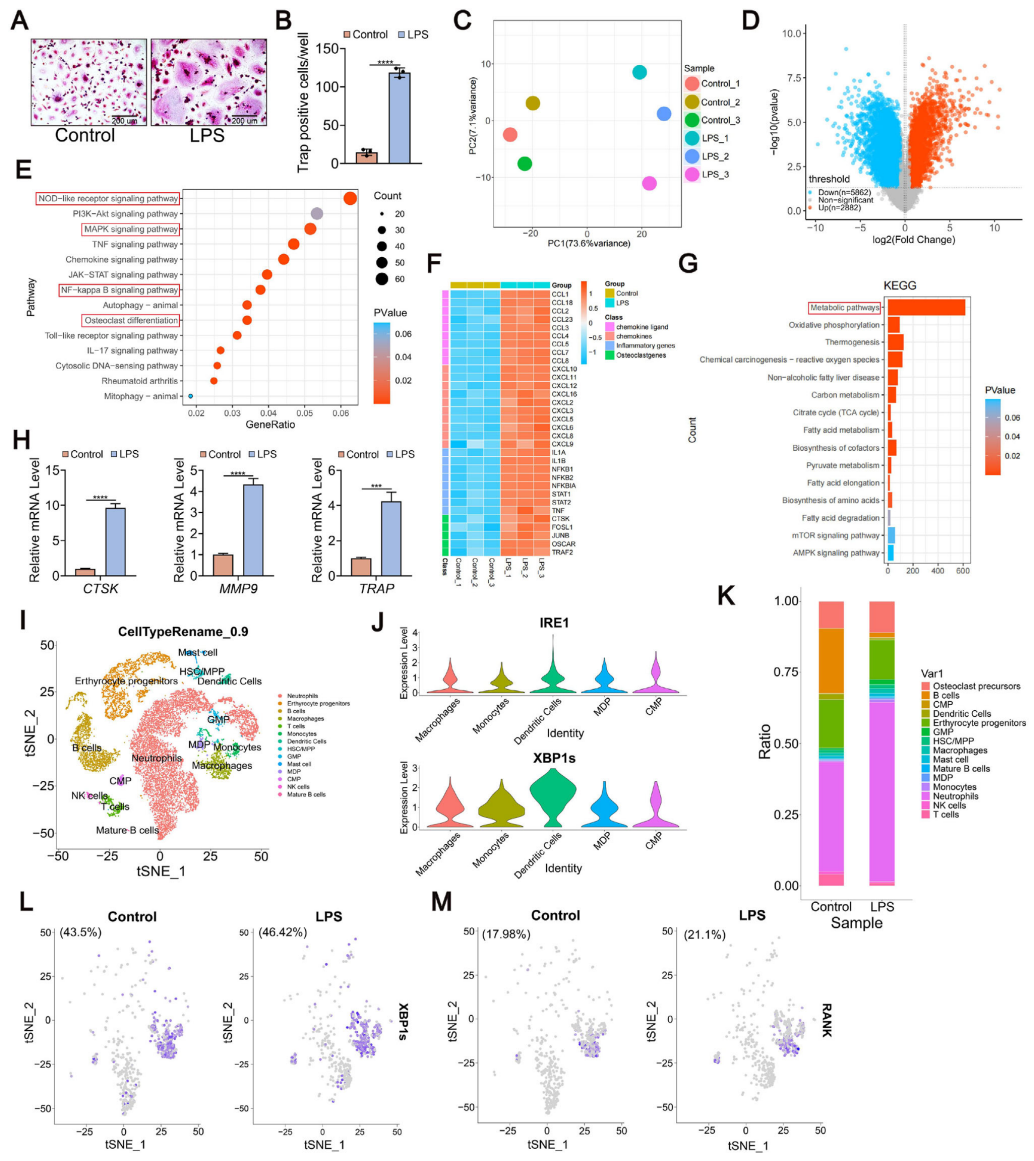
LPS induces human and mouse inflammatory osteoclastogenesis with the activation of IRE1‐XBP1s pathway. (A,B) Representative TRAP staining images (A) and quantification (B) of human osteoclasts induced from PBMCs with or without LPS stimulation. Scale bars, 200 µm. (C) PCA revealed different expression patterns in human PBMCs between the LPS‐treated group and control group. (D) Volcano plot shows DEGs between the two groups. (E) KEGG analysis of up‐regulated DEGs reveals that inflammatory and osteoclastogenic signaling pathways were activated. (F) The heatmap displays osteoclast genes and inflammatory genes increased by LPS. (G) KEGG analysis of down‐regulated DEGs shows that metabolic pathways were significantly related to inflammatory osteoclast differentiation. (I) The UMAP plot shows the main cell types of single‐cell RNA sequencing analysis. (J) Violin plots show the relative expression of IRE1 and XBP1s in the myeloid lineage of bone marrow cells. (K) The proportion of different cell types in the sham group and LPS group. (L) The proportion of XBP1s‐positive c‐Fms^+^ OCPs. (M) The proportion of XBPs‐positive and RANK‐positive c‐Fms^+^ OCPs. Data were presented as mean ± SEM, and the statistical significance was determined by two‐sided Student's test. Significance: ^*^ *p* < 0.05; ^**^ *p* < 0.01; ^***^ *p* < 0.001; ^****^ *p* < 0.0001.

In addition to mouse BMDMs, we isolated and cultured human peripheral blood mononuclear cells (PBMCs) in vitro and induced osteoclast differentiation. TRAP staining showed enhanced osteoclastogenesis after LPS treatment (Figure 1A,B). To gain more molecular insights on human inflammatory osteoclasts, we subjected LPS‐induced osteoclasts to RNA‐sequencing analysis. Principal components analysis revealed distinct transcriptional profiles between PBMCs and LPS osteoclasts (Figure 1C). Using a P value <0.05 and |log2(fold change)| >0.5, we identified 2,882 significantly upregulated and 5,862 downregulated differentially expressed genes (DEGs) following LPS stimulation (Figure 1D). Notably, KEGG pathway analysis showed significant enrichment of upregulated DEGs on NOD‐like receptor signaling pathways, which is the primary sensor for LPS‐induced inflammation. This enrichment was accompanied by activation of the MAPK and NF‐κB signaling pathways, which are well‐established regulators of osteoclast differentiation (Figure 1E). Consistently, LPS increased osteoclast marker genes and many inflammatory genes, such as chemokine ligands and chemokines (Figure 1F). Strikingly, KEGG pathway enrichment analysis of down‐regulated DEGs revealed predominant association with metabolic pathways, suggesting that LPS‐mediated promotion of human osteoclast differentiation may involve biochemical mechanisms related to intracellular metabolite regulation (Figure 1G). Further qPCR analysis confirmed the expression of osteoclast marker genes in LPS‐stimulated PBMCs (Figure 1H), in line with the findings seen in mouse osteoclasts.

### 3.2 The IRE1‐XBP1s Pathway is Activated During Inflammatory Osteoclast Differentiation

Inflammation osteoclast induced regional osteolysis is a major pathological feature of many inflammatory disorders and represents a promising therapeutic target for preventing bone destruction in these diseases. Our previous study has revealed the therapeutic potential of targeting the IRE1‐XBP1s pathway for treating osteoporosis, a systemic endocrine disorder [16]. However, the role of the IRE1‐XBP1s pathway in inflammatory osteolysis remains unknown. The IRE1‐XBP1s pathway is an important pathway in mediating the endoplasmic reticulum unfolded protein response (UPR), which is closely associated with the pathogenesis of inflammatory and neoplastic diseases [35, 36]. During inflammatory osteoclast differentiation, the IRE1‐XBP1s pathway is activated, as evidenced by increased phosphorylation level of IRE1 and time‐dependent increase of XBP1s after LPS treatment (Figure S1H,I). To further confirm the activation of this pathway in vivo, we challenged mice with LPS for 15 days and subjected the BMDMs to single‐cell RNA‐sequencing (Figure S1J). Osteoclasts derive from the myeloid lineage cells in the bone marrow. Using the t‐distributed Stochastic Neighbor Embedding (t‐SNE) dimensionality reduction technique, we identified 14 cell populations in the control and LPS groups, including cells of the myeloid lineage (Figure 1I). We defined osteoclast precursor cells (OCPs) as c‐Fms^+^ monocytes, macrophages, and dendritic cells, all of which express the cell surface marker and possess osteoclastogenic potential [37]. IRE1 and XBP1s were expressed in all the cell types of the myeloid lineage, suggesting potential involvement of the IRE1‐XBP1s pathway throughout osteoclastogenic lineage progression (Figure 1J). Cell composition analysis revealed higher proportion of OCPs in the LPS group (Figure 1K), accompanied by an increased proportion of neutrophiles and decreased lymphocytes. Notably, the proportion of XBP1s‐positive OCPs was significantly increased in LPS‐treated mice (Figure 1L), indicating activation of the IRE1‐XBP1s pathway within osteoclastogenic precursor populations during inflammatory stimulation. To further evaluate osteoclast lineage commitment, we analyzed the expression of RANK, a key receptor required for osteoclast differentiation. We found that the proportion of XBP1s‐positive OCPs co‐expressing RANK was markedly increased in the LPS group compared with controls (Figure 1M).

Collectively, these results demonstrate that LPS‐mediated bacterial inflammation promotes osteoclast differentiation and osteolysis, which may involve the activation of the IRE1‐XBP1s signaling pathway.

### 3.3 IRE1‐XBP1s Signaling Contributes to LPS‐Mediated Osteoclastogenesis and Osteolysis

**FIGURE 2:**
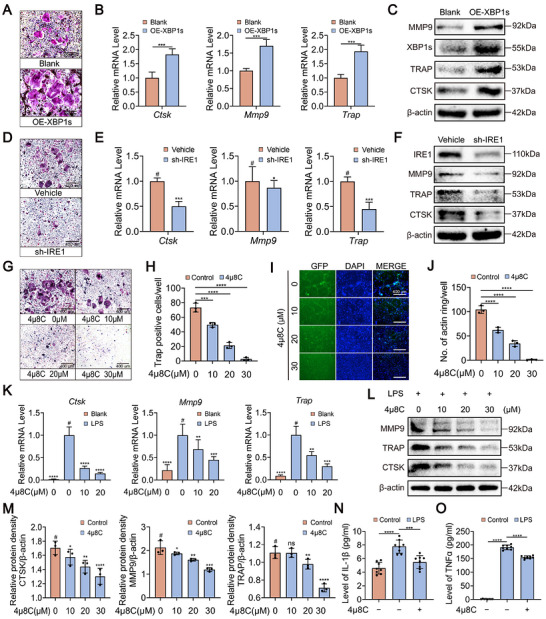
IRE1‐XBP1 signaling contributes to LPS‐mediated osteogenesis. (A–C) Overexpression of XBP1s promotes the differentiation of BMDMs into osteoclasts (A), accompanied by upregulated transcription (B) and translation (C) levels of osteoclast marker genes, including *Ctsk, Mmp9*, and *Trap*. (D‐F) Knockdown of IRE1 inhibits the differentiation of BMDMs into osteoclasts (D), accompanied by reduced mRNA expression (E) and translation (F) levels of osteoclast‐specific genes. (G,H) Representative TRAP staining images (G) and quantification (H) of osteoclasts induced from BMDMs with 4μ8C stimulation. Scale bars, 400 µm. (I,J) Representative fluorescence images (I) and quantification (J) of actin rings with 4μ8C stimulation. Scale bars: 400 µm. (K) 4μ8C attenuates the LPS‐induced upregulation of osteoclast marker gene mRNA expression. (L,M) The protein expression and quantitative analysis of Ctsk, Mmp9, and Trap with the treatment of 4μ8C. (N,O) 4μ8C inhibits the synthesis and secretion of pro‐inflammatory cytokines IL‐1β (N) and TNF‐α (O). Data were presented as mean ± SEM and the statistical significance was determined by two‐sided Student's test. Significance: ^*^ *p* < 0.05; ^**^ *p* < 0.01; ^***^ *p* < 0.001; ^****^ *p* < 0.0001.

To explore the role of IRE1‐XBP1s in LPS‐induced osteoclasts, we overexpressed XBP1s (OE‐XBP1s) in BMDMs via adenoviral trans‐infection and confirmed the overexpression efficiency (Figure S2A). As expected, the overexpression of XBP1s drastically enhanced inflammatory osteoclast differentiation (Figure 2A and Figure S2B) and increased the expression of osteoclast‐specific genes (Figure 2B). Western blotting analysis further revealed increased levels of osteoclast proteins in OE‐XBP1s osteoclasts (Figure 2C and Figure S2C). Original uncropped western blot images are provided in Figure S7. Thapsigargin (TG), a well‐established endoplasmic reticulum stress agonist, can significantly increase the phosphorylation of IRE1 (p‐IRE1) and upregulated XBP1s expression in a time‐dependent manner, indicating activation of the IRE1‐XBP1s signaling pathway following TG stimulation (Figure S2D–G). In addition to this genetic evidence, pharmacological activation of the IRE1‐XBP1s pathway with TG yielded similar promoting effects on LPS osteoclast differentiation (Figure S2H,I) and osteoclast gene expression (Figure S2J). Instead, we observed a significant reduction in LPS‐induced osteoclast formation following IRE1 knockdown using adenoviral shRNA (sh‐IRE1), and confirmed efficient gene silencing at an MOI of 200 (Figure 2D and Figure S2K,L). Knockdown of IRE1 also decreased osteoclast‐specific gene expression at the mRNA and protein level‌ (Figure 2E,F and Figure S2M). Consistent to IRE1 knockdown, pharmacological blocking of the IRE1‐XBP1s pathway with 4μ8C, a highly selective IRE1 inhibitor, dose‐dependently reduced the number of LPS‐osteoclasts (Figure 2G,H) and suppressed actin ring formation (Figure 2I,J), without showing evident cytotoxicity at these concentrations‌ (Figure S2N). The mRNA and protein levels of *Ctsk, Mmp9* and *Trap* were also decreased by 4μ8C treatment (Figure 2K–M). Furthermore, targeted inhibition of the IRE1‐XBP1s pathway by 4μ8C can also attenuate the pro‐osteoclastogenic effects of IL1β and TNFα (Figure S2O,P). In addition, ELISA analysis showed that 4μ8C decreased the secretion of IL‐1β and TNFα induced by LPS in osteoclasts (Figure 2N,O), indicating that targeting the IRE1‐XBP1s signaling is effective to suppression inflammatory osteoclastogenesis.

### 3.4 ‌Blocking the IRE1‐XBP1s Pathway Inhibits Inflammatory Bone Loss

**FIGURE 3:**
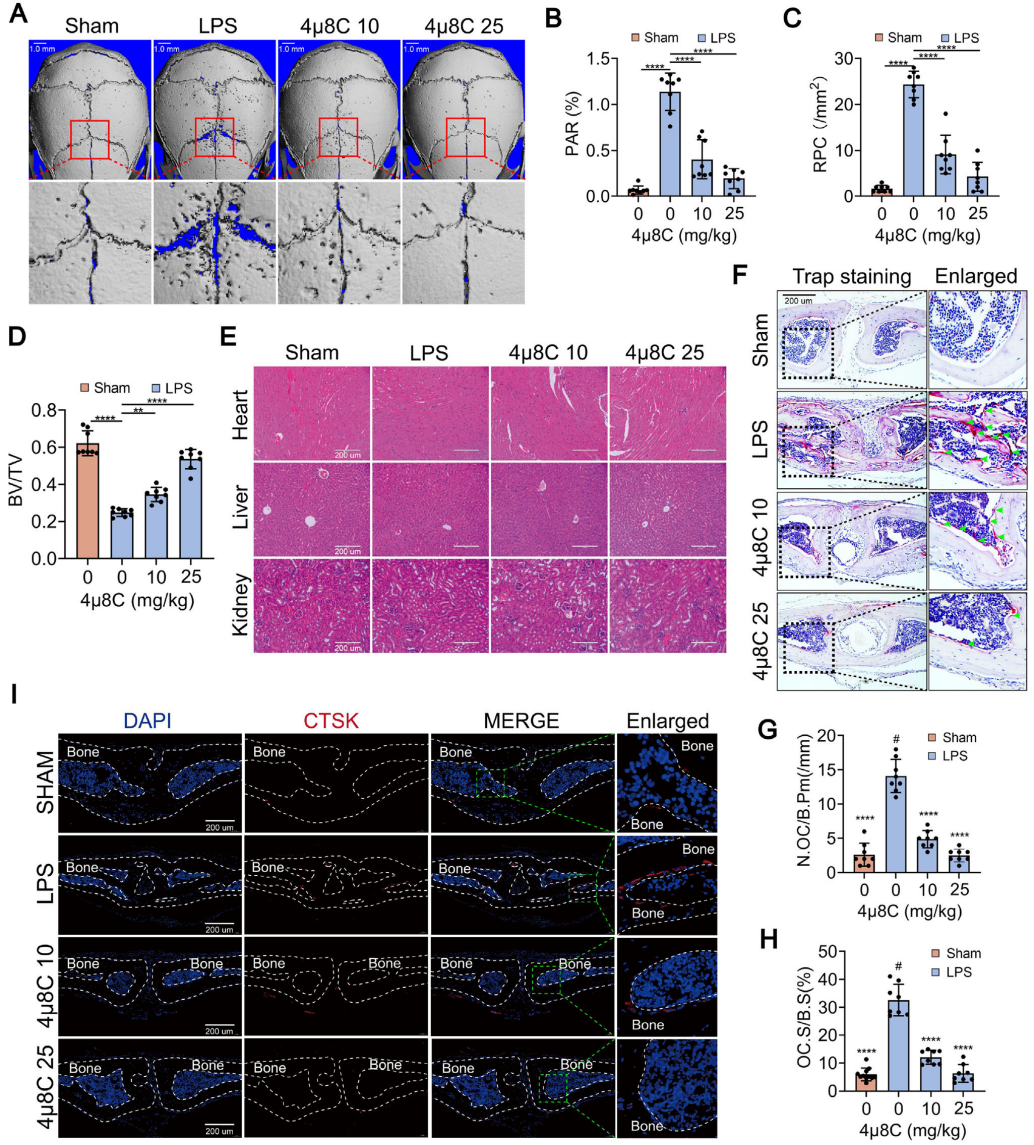
Blocking IRE1‐XBP1s pathway inhibits inflammatory bone loss. (A) Representative 3D reconstruction (micro‐CT) image of calvarium in each group. (B‐D) Quantification of PAR (resorption pit area ratio), RPC (Resorption pit count), and BV/TV (n ≥ 6). (E) H&E staining of the heart, liver, and kidney tissue in each group. Scale bars, 200 µm. (F) Representative TRAP staining images of calvarial slices from each group. Green arrows indicate osteoclasts. (G,H) Quantification of osteoclast‐related histomorphometric parameters N.OC/B.Pm and OC.S/B.S (n ≥ 6). (I) Immunofluorescence of CTSK‐labled calvarial slices from each group. Data were presented as mean ± SEM and the statistical significance was determined by two‐sided Student's test. Significance: ^*^ *p* < 0.05; ^**^ *p* < 0.01; ^***^ *p* < 0.001; ^****^ *p* < 0.0001.

We investigated whether blocking the IRE1‐XBP1s signaling pathway could protect against LPS‐induced inflammatory bone loss in vivo. LPS‐treated mice received daily intraperitoneal administration of low‐dose (10 mg/kg) and high‐dose (25 mg/kg) 4μ8C for 15 days [38, 39]. µCT scanning revealed overt bone erosion of mice in the LPS group (Figure 3A), which was further evidenced by increased bone erosion area and more erosion pits in the cranial suture area. However, 4μ8C treatment dose‐dependently suppressed the bone erosion (Figure 3B–D). H&E staining of heart, liver, and kidney tissues revealed no evident organ toxicity at the administered doses of 4μ8C‌ (Figure 3E). Then, TRAP staining‌ on coronal sections of the skull revealed enhanced osteoclast differentiation after LPS treatment, which was reversed by 4μ8C treatment (Figure 3F). Histomorphometric analysis revealed decreased osteoclast number and osteoclast surface area after ‌4μ8C treatment (Figure 3G,H). ‌CTSK is the coreexecutor‌ of osteoclastic boneresorption‌ through direct degradation of the bone matrix. In vivo, immunofluorescence revealed elevated CTSKexpression‌ in cranial bones of LPS‐treated mice, but was dose‐dependentlysuppressed‌ by 4μ8C (Figure 3I). Meanwhile, toluidine blue staining and histomorphometric analysis revealed no significant differences in osteoblast‐related parameters among the experimental groups (Figure S3A–C). Consistently, serum P1NP levels were unchanged following 4μ8C treatment, suggesting that its protective effect against bone erosion is unlikely to be mediated through enhanced bone formation (Figure S3D). These findings reveal that pharmacological inhibition of the IRE1‐XBP1s signaling pathway with 4μ8C effectively suppresses LPS‐induced bone erosion in vivo.

### 3.5 IRE1‐XBP1s Signaling Pathway Regulates Slc6a4 Expression

**FIGURE 4:**
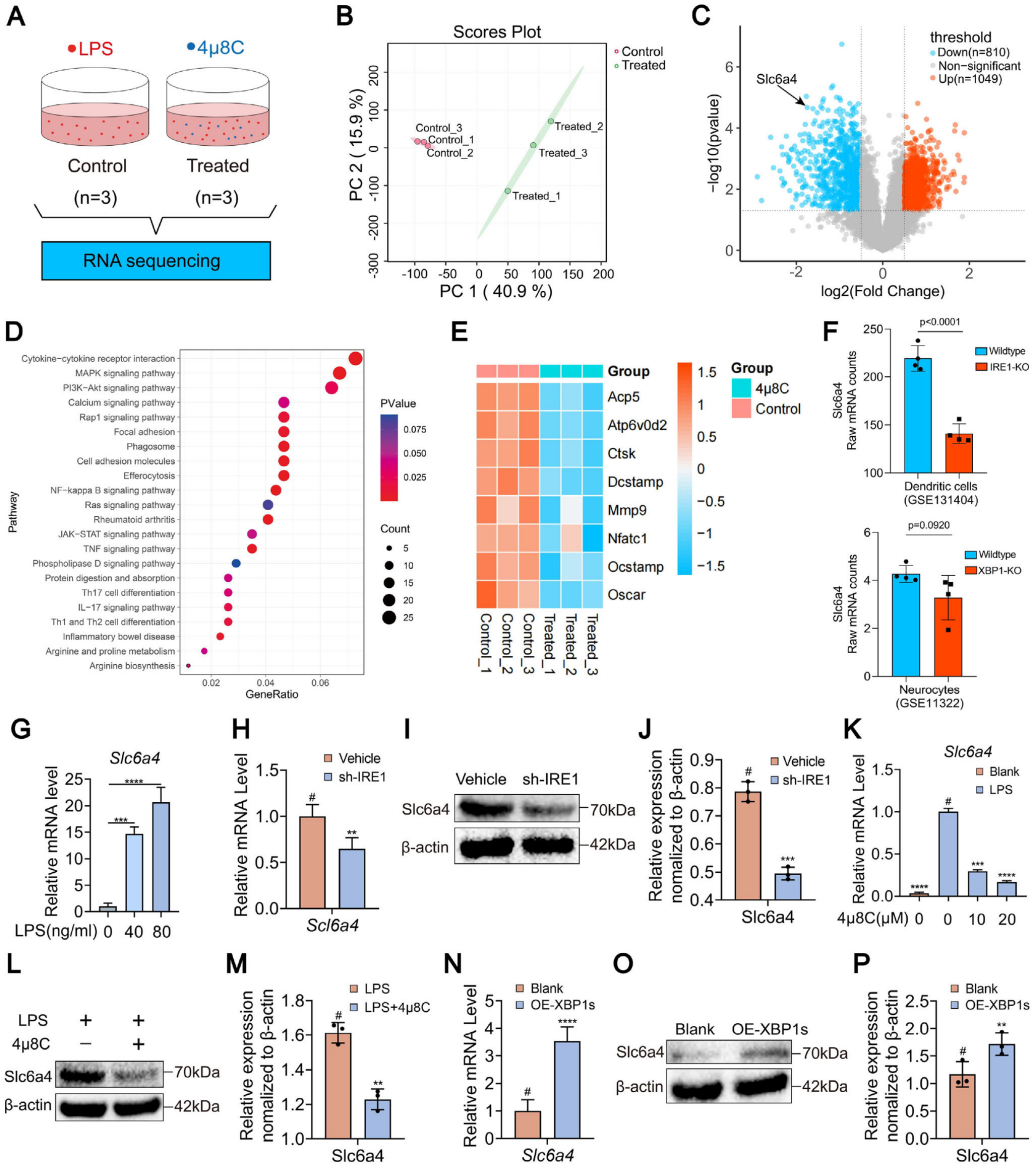
IRE1‐XBP1s signaling regulates Slc6a4 gene expression. (A) The design of the RNA sequencing assay with the treatment of 4μ8C. (B) The principal component analysis (PCA) of the RNA‐sequencing data between the control and 4μ8C‐treated groups. (C) Volcano plot showing the differentially expressed genes between the two groups. (D) KEGG pathway analysis of the 810 4μ8C‐decreased genes. (E) The heatmap showing the changes of osteoclast‐specific genes between the two groups. (F) GSE database analysis reveals changes in Slc6a4 expression following IRE1 or XBP1s knockout in dendritic cells and neurocytes, respectively. (G) qPCR shows the mRNA expression of Slc6a4 with LPS stimulation in a dose‐dependent manner. (H–J) Knockdown of IRE1 in BMDMs via adeno‐shRNA attenuates Slc6a4 expression at mRNA and protein levels, as demonstrated by quantitative analysis (*n* = 3). (K–M) 4μ8C attenuates Slc6a4 expression at both mRNA and protein levels, as demonstrated by quantitative analysis (*n* = 3). (O,P) Overexpression of XBP1s in BMDMs promotes Slc6a4 expression at the mRNA and protein levels, as demonstrated by quantitative analysis (*n* = 3). Data were presented as mean ± SEM and the statistical significance was determined by two‐sided Student's test. Significance: ^*^ *p* < 0.05; ^**^ *p* < 0.01; ^***^ *p* < 0.001; ^****^ *p* < 0.0001.

To identify downstream targets of the IRE1‐XBP1s signaling pathway in inflammatory osteoclasts, we treated BMDMs with 4μ8C and performed RNA sequencing analysis (Figure 4A). The overall transcriptional pattern was distinct between the control and 4μ8C‐treated groups (Figure 4B). Using a P‐value < 0.05 and |log2 (Fold change)| > 0.5 as the screening criteria, we identified 810 downregulated genes in the 4μ8C‐treated group (Figure 4C). KEGG analysis revealed that these downregulated genes were enriched in the MAPK, PI3K‐Akt, and NF‐κB signaling pathways (Figure 4D), which are canonical pathways involved in osteoclast differentiation and survival [40, 41, 42]. Notably, we found *Slc6a4* was among the most downregulated genes, and the downregulation of this gene was accompanied by decreased expression of osteoclast marker genes, including *Trap*, *Mmp9*, and *Ctsk* in the 4μ8C groups (Figure 4E). Additionally, public GEO datasets also showed decreased gene expression of *Slc6a4* in dendritic cells from IRE1 knockout mice (GSE131404) and in neurocytes from XBP1s knockout mice (GSE11322) (Figure 4F), indicating that *Slc6a4* may be a downstream of the IRE1‐XBP1s signaling pathway.

PCR assay revealed increased expression of *Slc6a4* by LPS in osteoclasts (Figure 4G). To confirm *Slc6a4* is the downstream gene of IRE1‐XBP1s in LPS osteoclasts, we silenced IRE1 gene expression via adeno‐shRNA in LPS‐stimulated BMDMs (Figure 4H). Knockdown of IRE1 in BMDM decreased the protein expression of *Slc6a4* (Figure 4I–J). Consistent to this result, pharmacological IRE1‐XBP1s signaling inhibition also decreased the mRNA and protein levels of *Slc6a4* (Figure 4K–M). Conversely, we overexpressed XBP1s in LPS‐stimulated BMDMs and found increased *Slc6a4* expression at the mRNA and protein levels (Figure 4N–P), indicating that *Slc6a4* is a downstream gene regulated by the IRE1‐XBP1s pathway.

### 3.6 ‌‌‌XBP1s Functions as a Transcription Factor That Specifically Targets and Regulates Slc6a4 Gene Expression‌

**FIGURE 5:**
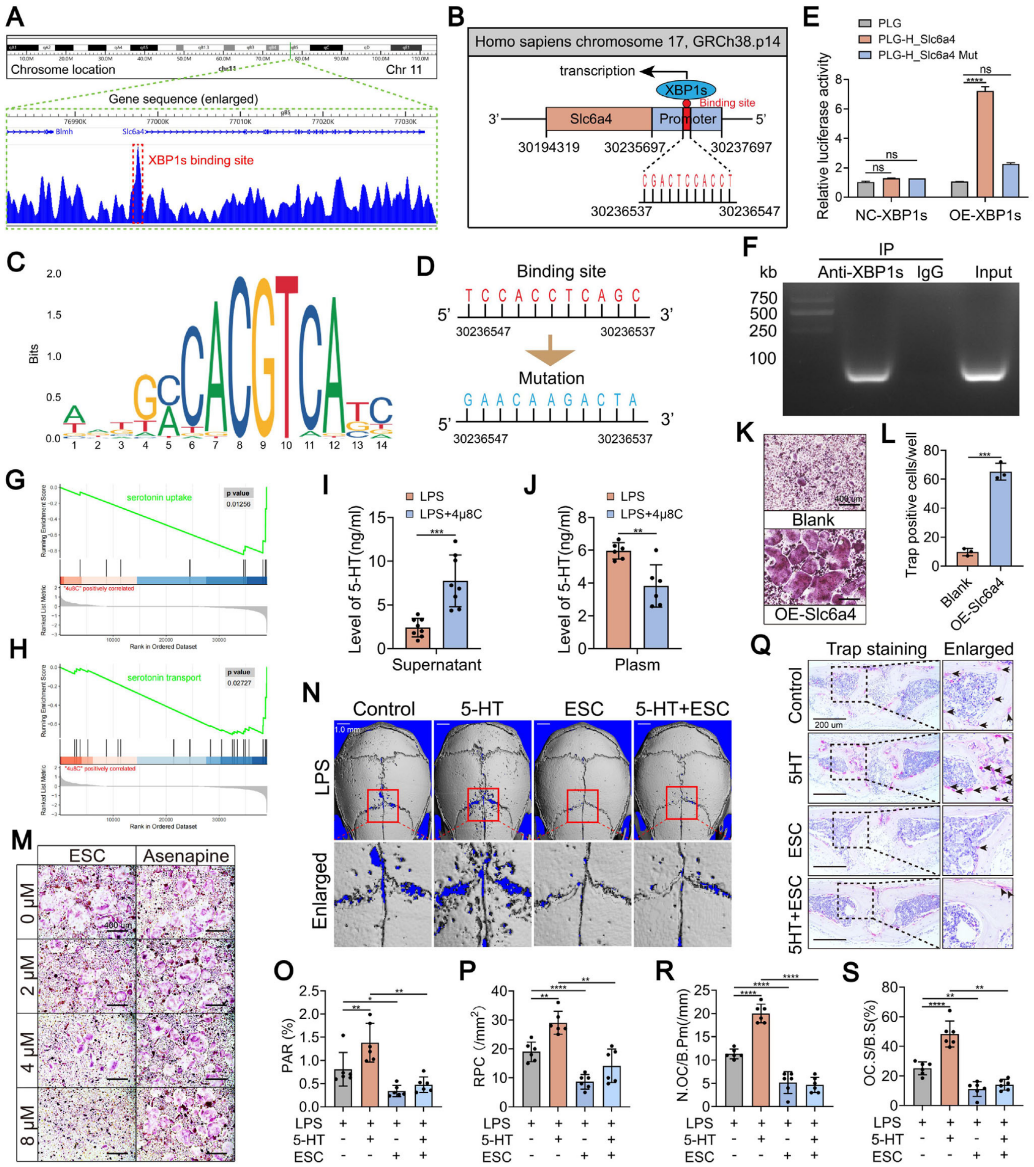
XBP1s activates the transcription of the 5‐HT transporter Slc6a4. (A) The chromosomal location, gene sequence, and the predicted XBP1s binding site within the promoter region of murine Slc6a4. (B) The schematic diagram of the chromosomal location, gene structure, and the predicted XBP1s binding site within the promoter region of human Slc6a4. (C) The binding motif of human XBP1s obtained from the JASPAR database. (D) The wild‐type and mutant sequence of the predicted human XBP1s binding site in the luciferase reporter constructs. (E) Dual luciferase activity of blank and overexpression human XBP1s in the wild‐type and mutant Slc6a4 promoter groups (*n* = 3 per group). (F) ChIP‐qPCR results showing the binding of XBP1s to the promoter of Slc6a4. Homologous IgG antibody was used as a negative control in the ChIP assay, and the input lysis was used as the positive control in the PCR analysis. (G) GSEA of the 5‐HT uptake pathway between the control and 4μ8C‐treanted groups. (H) GSEA of the 5‐HT transport pathway between the control and 4μ8C‐treanted groups. (I) The level of 5‐HT in the supernatant of culture medium in the LPS and 4μ8C‐treanted groups (*n* = 8 per group). (J) Intracellular 5‐HT levels in the LPS and 4μ8C‐treanted groups (*n* = 6 per group). (K,L) Representative TRAP staining images (K) and quantification (L) of osteoclasts induced from BMDMs with Slc6a4 overexpression. Scale bars, 400 µm. (M) The escitalopram (5‐HT transporter inhibitor) and asenapine (5‐HT receptor inhibitor) treatment on LPS‐mediated osteoclast differentiation in a similar concentration gradient manner. (N) Representative 3D reconstruction (micro‐CT) image of calvarium in each group. (O‐P) Quantification of bone mass parameters PAR (resorption pit area ratio) and RPC (Resorption pit count) (*n* = 6 per group). (Q) Representative Trap staining images of calvarial slices from each group. Black arrows indicate osteoclasts. (R,S) Quantification of osteoclast‐related histomorphometric parameters N.OC/B.Pm and OC.S/B.S (*n* = 6). Data were presents as mean ± SEM and the statistical significance was determined by two‐sided Student's test. Significance: ^*^ *p* < 0.05; ^**^ *p* < 0.01; ^***^ *p* < 0.001; ^****^ *p* < 0.0001.

We then explored how the IRE1‐XBP1s pathway regulates Slc6a4 expression. XBP1s is an active transcription factor activated by IRE1 during unfold protein response. A public Chip‐seq dataset (ID: 73674) in mouse BMDMs showed response peaks to XBP1s in the promoter region of the Slc6a4 gene (Figure 5A). We speculated that Slc6a4 is a direct transcriptional target of XBP1s. The JASPAR database showed a potential XBP1s binding site in the promoter region of the human Slc6a4 gene (Figure 5B). The sequence of the predicted binding site, located at ‐578∼‐565 upstream of the transcriptional starting site, aligned with the binding motif of XBP1s (Figure 5C).‌ To confirm that XBP1s activates Slc6a4 expression by binding to this site, we constructed pGL3‐basic luciferase reporter plasmids containing the putative site mutant promoter sequence before the luciferase gene (Figure 5D). We co‐transfected the mutant plasmids into 293T cells along with XBP1s‐overexpressing plasmids and pRL‐TK plasmids. Luciferase assay using the renilla luciferase as reference showed that the luciferase activity was enhanced by the wild‐type Slc6a4 promoter, whereas a mutation in the putative sequence attenuated this activation (Figure 5E). In addition, ChIP‐PCR assay confirmed the binding of XBP1s to the promoter regions using primers targeting the −1,837–−1,817 region (Figure 5F). These results indicated that XBP1s binds to the promoter of the Slc6a4 gene and activates its expression.

The Slc6a4 gene encodes the 5‐hydroxytryptamine transporter (5‐HTT), which mediates serotonin/5‐hydroxytryptamine (5‐HT) uptake by cells from extracellular space. We investigated whether the IRE1‐XBP1s axis modulates 5‐HT uptake during LPS osteoclastogenesis. GSEA analysis on the 4μ8C RNA‐seq data revealed significant enrichment of 5‐HT uptake and 5‐HT transport pathways following inhibition of the IRE1‐XBP1s pathway (Figure 5G,H). Consistent to decreased Slc6a4 expression, ‌an ELISA assay demonstrated increased 5‐HT concentration in the culture supernatant and decreased intracellular 5‐HT levels in osteoclasts after 4μ8C treatment, indicating decreased 5‐HT uptake by LPS osteoclasts (Figure 5I,J).

To further determine whether Slc6a4 functionally contributes to osteoclast differentiation, we overexpressed Slc6a4 in BMDMs using an adenoviral vector. Efficient overexpression was confirmed by qPCR analysis, which demonstrated a significant increase in Slc6a4 mRNA expression compared with the Blank control group (Figure S4A). Importantly, Slc6a4 overexpression significantly increased the transcription levels of osteoclast marker genes (Figure S4B). Consistent with these findings, TRAP staining revealed a marked increase in the number of multinucleated osteoclasts in the OE‐Slc6a4 group compared with the control group (Figure 5K,L). These results indicate that Slc6a4 actively promotes osteoclast differentiation and support its role as a functional downstream effector of the IRE1‐XBP1s pathway during inflammatory osteoclastogenesis.

‌5‐HT functions as a neurotransmitter on postsynaptic neurons, but also circulates in the blood to act on other tissues and organs throughout the body. However, the role of 5‐HT on inflammatory osteoclasts remains largely unknown. LPS induced osteoclast differentiation was enhanced in a dose‐dependent manner after exogenously supplementing 5‐HT to the culturing medium (Figure S4C,D). In the body, 5‐HT exerts its function by either acting on the receptors in the plasma membrane or by entering into cells via transmembrane transporters. To explore how 5‐HT facilitates inflammatory osteoclast formation, we treated cells with escitalopram, a selective serotonin reuptake inhibitor, and asenapine, a 5‐HT receptor antagonist. TRAP staining revealed robust suppression on inflammatory osteoclasts differentiation by escitalopram, but not asenapine (Figure 5M and Figure S4E,F). Escitalopram also downregulated osteoclast‐specific markers under LPS stimuli (Figure S4G), indicating that 5‐HT promotes LPS osteoclasts within the cells, rather than acting on its receptors. Escitalopram and asenapine showed no cytotoxicity on LPS osteoclasts at the used concentrations (Figure S4H,I). In vivo, intraperitoneal administration of 5‐HT exacerbated calvarial bone destruction, increased bone erosion area and resorption pits, whereas escitalopram alleviated inflammatory bone erosion and counteracted the effects of 5‐HT (Figure 5N–P). Histopathological ‌H&E staining sections‌ revealed ‌no evident organ toxicity‌ at the employed doses of 5‐HT and escitalopram on the heart, liver, and kidney in the experimental mice‌ (Figure S4J). TRAP staining and histomorphometric analysis showed increased osteoclast formation and higher N.OC/B.Pm and OC.S/B.S in the calvaria of mice after 5‐HT treatment, whereas escitalopram reversed these effects (Figure 5Q–S), suggesting that inhibition of 5‐HT uptake by escitalopram suppresses inflammatory osteoclast formation and alleviates LPS‐induced bone erosion. Meanwhile, osteoblast formation was not changed by 5‐HT and ESC (Figure S4K–M).

### 3.7 5‐HT Facilitates Inflammation Response and Decreases the Intracellular Level of an Anti‐Autophagy Compound

**FIGURE 6:**
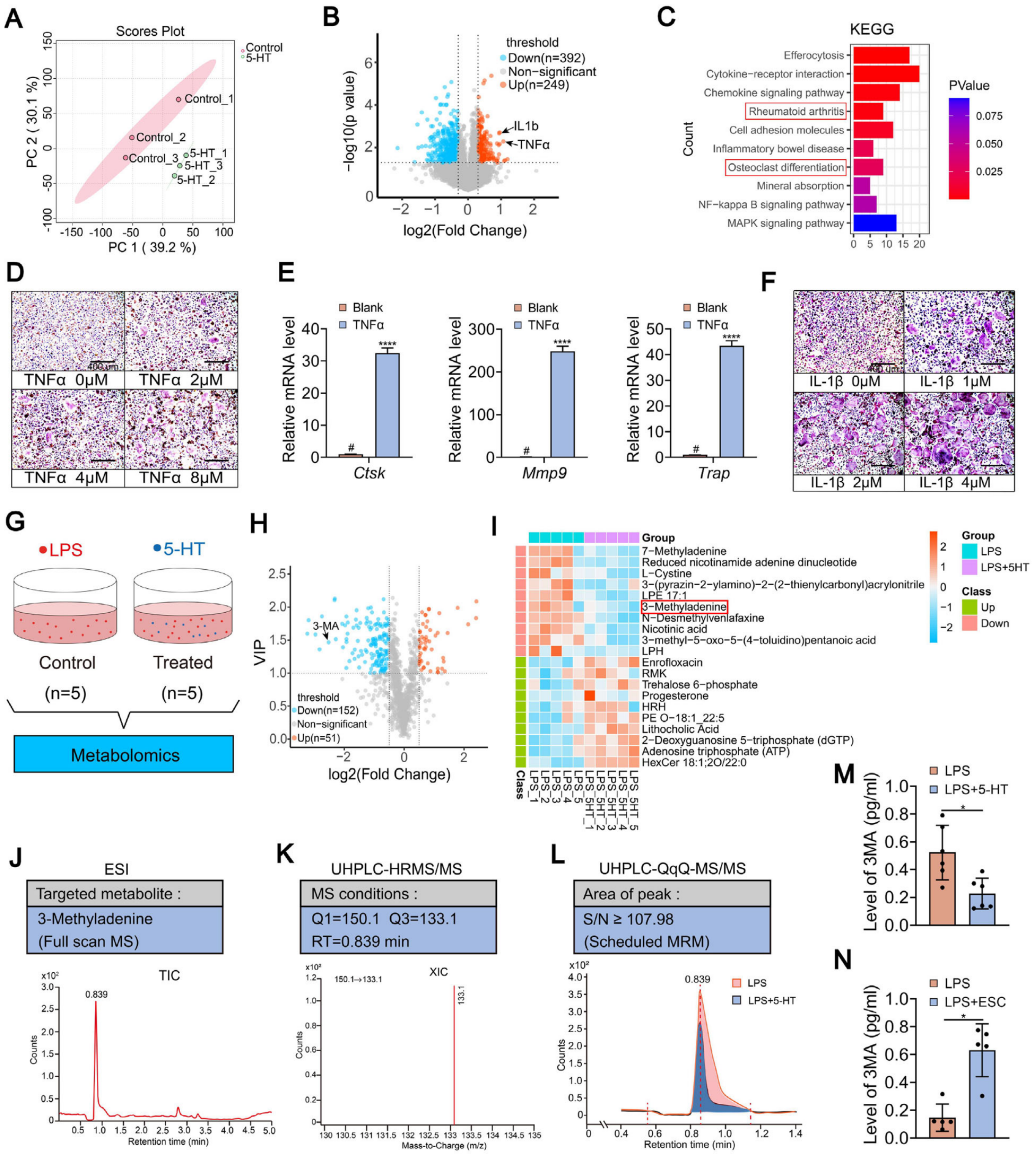
5‐HT promotes LPS‐mediated osteoclast differentiation through endogenic anti‐autophagy mechanism. (A) The principal component analysis of the RNA‐sequencing data in the control and 5‐HT‐treated groups. (B) Volcano plot shows that 5‐HT upregulates 249 DEGs, including IL‐1β and TNFα. (C) KEGG pathway analysis of the 249 5‐HT‐upregulated genes. (D) Representative TRAP staining images of mature osteoclasts with different TNFα stimulation. Scale bars: 400 µm. (E) mRNA expression of osteoclast‐associated genes in BMDMs with or without TNFα stimulation. (F) Representative TRAP staining images of mature osteoclasts with different IL‐1β stimulation. Scale bars: 400 µm. (G) The design of non‐targeted metabolomics analysis with or without the treatment of 5‐HT. (H) Volcano plot shows 152 reduced metabolites with the intervention of 5‐HT. (I) The heatmap displays primarily downregulated and upregulated metabolites under 5‐HT treatment. (J) The total ion chromatogram (TIC) displays the retention time (RT) of the 3MA standard peak under multiple reaction monitoring (MRM), used to evaluate the mass spectrometry (MS) conditions. (K) The mass‐to‐charge ratio (m/z) range and RT for 3MA were set as the MS conditions, under which 3MA could be clearly detected with observable peak formation in the extracted ion chromatogram (EIC). (L) In the EIC, the peak area of 3MA showed a linear correlation with its standard concentration (R = 0.9922), and the signal‐to‐noise ratio (S/N) was ≥107.98, meeting the quantitative analysis requirements for experimental samples. (M) Quantitative analysis shows the mass spectrometry detection results of intracellular 3MA with or without 5‐HT stimulation (N = 6). (N) Mass spectrometry detection and quantitative analysis of intracellular 3MA levels with or without escitalopram stimulation (N = 5). Data were presented as mean ± SEM, and the statistical significance was determined by two‐sided Student's test. Significance: ^*^ *p* < 0.05; ^**^ *p* < 0.01; ^***^ *p* < 0.001; ^****^ *p* < 0.0001.

The mechanism by which intracellular 5‐HT facilitates inflammatory osteoclast differentiation remains unclear. Therefore, we combined RNA‐seq and untargeted metabolomics to explore the mechanism. Compared to the control group, 5‐HT stimulation greatly changed the transcription pattern of inflammatory osteoclasts (Figure 6A). Volcano plot analysis identified 249 upregulated genes upon 5‐HT treatment, including the proinflammatory factor TNFα and IL‐1β (Figure 6B). Accordingly, these upregulated genes were enriched in osteoclast differentiation and many inflammatory pathways by KEGG analysis (Figure 6C). In vitro osteoclast differentiation assays showed enhanced osteoclast differentiation after TNFα stimulation (Figure 6D and Figure S5A), accompanied by markedly enhanced expression of osteoclast‐specific genes (Figure 6E). Similar promoting effects were observed after IL‐1β stimulation (Figure 6F and Figure S5B), indicating that 5‐HT is a potent inflammation driver to promote osteoclast differentiation.

5‐HT has been reported to regulate diverse metabolic processes [43]. Interestingly, our above KEGG analysis revealed that metabolic processes were also the most profound pathway in LPS‐induced osteoclasts (Figure 1G). To investigate whether 5‐HT enhances inflammatory cytokine production and osteoclast differentiation via metabolic mechanisms, we performed untargeted metabolomic profiling on LPS‐treated osteoclasts with or without 5‐HT stimulation (Figure 6G). Using variable importance in projection (VIP) > 1 and |log2(Fold change)| > 0.5, we identified 152 downregulated metabolites and 51 upregulated metabolites in 5‐HT stimulated LPS osteoclasts (Figure 6H). Among the downregulated metabolites, we were intrigued by 3‐methyladenine (3MA), which was among the top 10 significantly altered metabolites (Figure 6I). 3MA is conventionally recognized as an artificially synthesized autophagy inhibitor, and no studies have reported its presence in mammalian cells. To avoid potential false positive inherent in untargeted metabolomics, we employed High Performance Liquid Chromatography‐Mass Spectrometry (HPLC‐MS) to confirm the presence of 3MA in LPS osteoclasts. We established a standard curve using artificially synthesized 3MA as the reference standard at serial concentrations, with Retention Time (RT) = 0.836 min defined as the key mass spectrometry parameter. Both standards and test samples exhibited overt peaks at RT of 0.836 min, characterized by significantly enhanced ion response intensity (Figure 6J). During mass spectrometry detection, the mass‐to‐charge ratios (m/z) for the primary precursor ion (Q1) and secondary product ion (Q3) of 3MA were set at 150.1 and 133.1, respectively. Both ions were captured within the target chromatographic peak at RT = 0.836 min, with their elevated ion intensities providing strong evidence for the presence of 3MA. A representative ion chromatogram from the control group is presented (Figure 6K). To quantitatively analyze the target metabolite 3MA, we first validated its robust signal intensity with a measured signal‐to‐noise ratio (SNR) ≥ 107.98 across all samples—significantly exceeding the conventional lower limit of quantification (LLOQ) threshold of SNR ≥ 10. Subsequent quantification of the target ion peak utilized the area under the curve (AUC), where larger AUC values correlate with higher metabolite concentrations (Figure 6L). Cross‐referencing these AUC values against the calibration curve enabled precise concentration determination. After confirming the presence of 3MA in osteoclasts, we detected the intracellular concentration of 3MA using HPLC‐MS and found that 5‐HT significantly decreased 3MA in osteoclasts (Figure 6M), confirming the results observed by the untargeted metabolomics. On the contrary, blocking 5‐HT uptake with escitalopram significantly elevated intracellular 3MA level in osteoclasts (Figure 6N).‌ Collectively, these results revealed that artificially synthesized 3MA also presents naturally in inflammatory osteoclasts and that the intracellular concentration of this metabolite is affected by 5‐HT.

### 3.8 3MA Suppresses 5‐HT‐Mediated Enhancement of Cellular Autophagy

**FIGURE 7:**
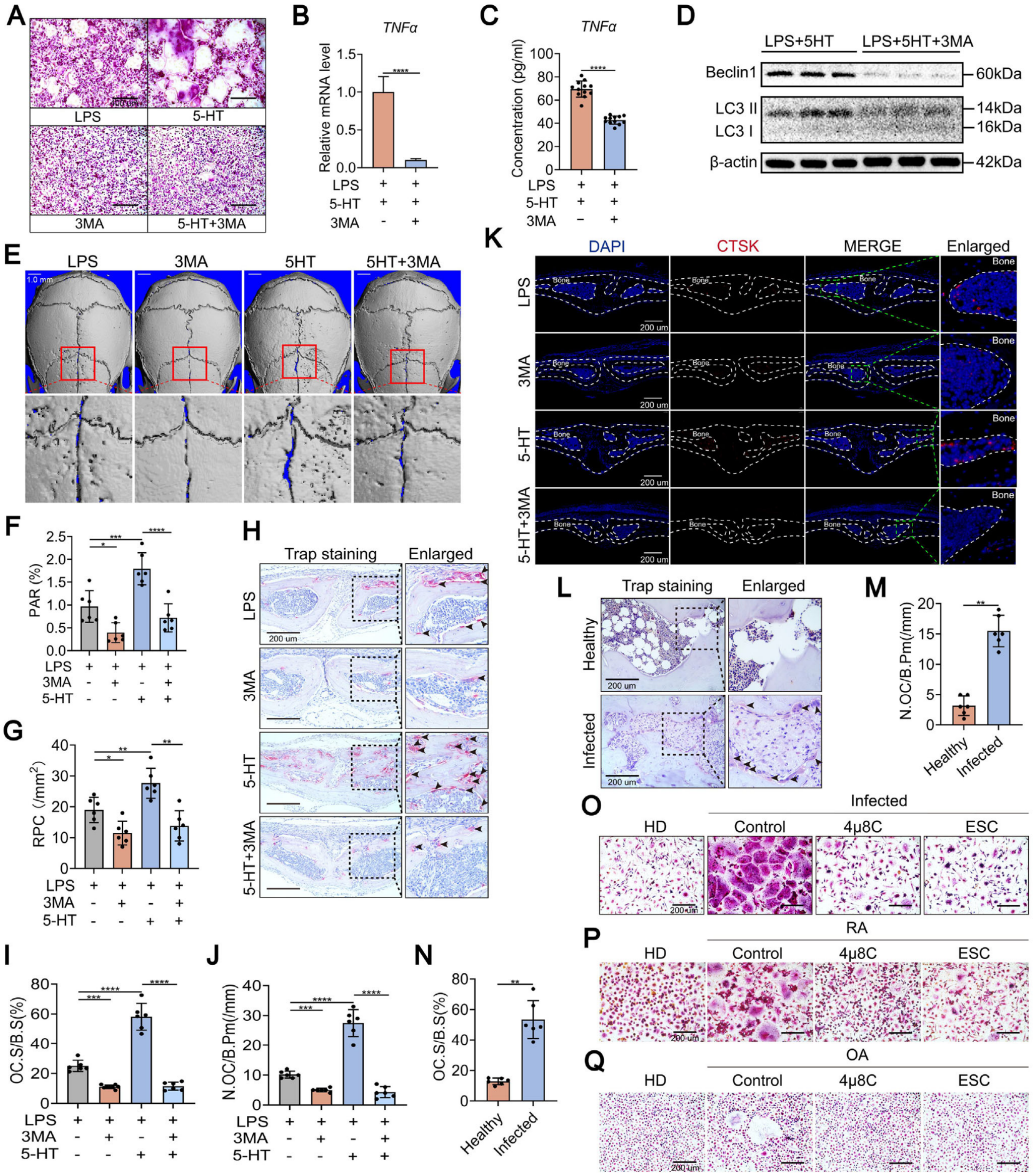
3MA suppresses 5‐HT‐mediated enhancement of cellular autophagy. (A) Representative TRAP staining images of mature osteoclasts with or without 5‐HT and 3MA stimulation. Scale bars: 400 µm. (B) qPCR result shows the expression of TNFα with the supplementation of 3MA. (C) ELISA analysis shows the TNFα level in the culture medium supernatant. (D) Protein levels of autophagy‐special genes in the BMDMs with or without the supplementation of 3MA. (E) Representative 3D reconstruction (micro‐CT) images of calvarium in each group. (F,G) Quantification of bone mass parameters PAR (F) and RPC (G) (n = 6 per group). (H) Representative Trap staining images of calvarial slices from each group. Black arrows indicate osteoclasts. (I,J) Quantification of osteoclast‐related histomorphometric parameters OC.S/B.S and N.OC/B.Pm (*n* = 6 per group). (K) Immunofluorescence of CTSK‐labeled calvarial slices from each group. (L‐N) Representative images of TRAP staining (L) and quantification of PAR (M) and RPS (N) in healthy donors and myelitis patient groups (*n* = 6 per group). Scale bars: 200 µm. (O–Q) Representative TRAP staining images in mature human osteoclasts induced from peripheral blood mononuclear cells (PBMC) of healthy donors, myelitis (O), RA (P), and OA (Q) patients. Scale bars: 200 µm. Data were presented as mean ± SEM, and the statistical significance was determined by two‐sided Student's test. Significance: ^*^ *p* < 0.05; ^**^ *p* < 0.01; ^***^ *p* < 0.001; ^****^ *p* < 0.0001.

‌3MA is an autophagy inhibitor, and we then investigated whether this metabolite affects autophagy in inflammatory osteoclasts. LPS‐induced osteoclast differentiation was dose‐dependently suppressed by 3MA at its non‐toxic concentrations (Figure S6A–C). 3MA also abolished the promoting effect of 5‐HT on LPS osteoclasts (Figure 7A).‌ This inhibitory effect was accompanied by reduced production of inflammatory cytokines, as qPCR and ELISA assays revealed decreased TNFα expression even in the presence of 5‐HT (Figure 7B,C). Consistent with its conventional function, 3MA reduced the expression of autophagy‐related markers, including Beclin1, and decreased the LC3‐II/LC3‐I ratio (Figure S6D).‌ Western blot confirmed that 3MA inhibited the synthesis of autophagy‐related proteins (Figure 7D and Figure S6E). Furthermore, 3MA markedly attenuated TG‐induced osteoclast differentiation, supporting a downstream role of 3MA‐associated autophagic regulation in IRE1‐XBP1s mediated osteoclastogenesis (Figure S6F,G). ‌In vivo, 3MA treatment significantly alleviated LPS‐induced osteolysis and attenuated the aggravation of bone erosion induced by 5‐HT (Figure 7E). µCT and bone resorption area analyses further confirmed the inhibitory effect of 3MA on inflammatory bone loss (Figure 7F,G). H&E staining precluded cardiac, hepatic, and renal toxicity of 3MA at the employed doses (Figure S6H).‌ Histomorphometric analysis and TRAP staining of calvarial sections revealed that 3MA reversed the osteoclast‐promoting effect of 5‐HT in vivo (Figure 7H–J), whereas osteoblast formation was not changed by 5‐HT and 3MA (Figure S6I–K). Accordingly, immunofluorescence assay revealed decreased expression of osteoclast CTSK expression by 3MA treatment in the 5‐HT treated LPS mice (Figure 7K), indicating that 3MA, as an endogenous autophagy inhibitor, is effective in ameliorating inflammatory bone loss.‌

### 3.9 Targeting the IRE1‐XBP1s‐Slc6a4 Pathway Suppressed Inflammatory Osteoclast From Patients With Inflammatory Diseases

‌In this section, we explore the effects of targeting the IRE1‐XBP1s‐Slc6a4 pathway with on inflammatory osteoclasts from human patients.‌ ‌For this evaluation, we used the IRE1‐XBP1s inhibitor 4μ8C or the 5‐HT uptake inhibitor escitalopram. First, we obtained human lumbar spine specimens from patients with gram‐negative osteomyelitis and used specimens from patients with etrauma‐induced comminuted fractures as the control group. Histological TRAP staining revealed increased osteoclast formation at the infectious bones, accompanied by pronounced inflammatory infiltration and marked adipocyte depletion within marrow cavities (Figure 7L–N). In vitro osteoclastogenesis assays demonstrated that PBMCs from the osteomyelitis patient exhibited hyperresponsiveness to Human RANKL stimulation (50 ng/mL), generating more TRAP staining positive multinucleated cells compared to fracture patient‐derived PBMCs. Notably, pharmacological intervention with either the IRE1 inhibitor 4μ8C or selective 5‐HT reuptake inhibitor escitalopram suppressed PBMCs‐derived osteoclast differentiation (Figure 7O and Figure S6L), suggesting their potential therapeutic utility in infection‐associated bone resorption in humans. Given that rheumatoid arthritis (RA) and osteoarthritis (OA) represent prevalent chronic sterile inflammatory disorders, we also investigated the impact of blocking the IRE1‐XBP1s‐Slc6a4 signaling axis on osteoclastogenesis in both conditions. Notably, PBMCs from RA and OA patients exhibited significantly enhanced in vitro osteoclast differentiation capacity compared to healthy controls, but the pharmacological inhibition with either 4μ8C or escitalopram synergistically attenuated inflammation‐driven osteoclast formation (Figure 7P,Q and Figure S6M,N). We have included the clinical information of all enrolled patients in Supplementary Table 1. These findings showed that targeting the IRE1‐XBP1s‐Slc6a4 pathway with either IRE1‐XBP1s inhibitor or a 5‐HT uptake inhibitor represents a promising strategy for the treatment of inflammatory osteolysis diseases.

## 4 Discussion

Given the substantial health burden associated with inflammatory bone loss and the lack of therapies specifically targeting inflammatory osteoclasts, further investigation into the molecular mechanisms underlying inflammation‐induced osteoclast differentiation and bone resorption is essential for identifying new therapeutic targets. The present study elucidates the critical role of the IRE1‐XBP1s axis in LPS‐mediated inflammatory osteoclastogenesis and osteolysis, revealing a novel mechanistic link among ER stress, 5‐HT transport, and autophagy regulation in bone resorption. Our findings not only expanded the current understanding of inflammatory bone loss but also provide potential therapeutic targets for treating bacterial and sterile inflammatory bone diseases.

Human PBMCs are a population of mononuclear leukocytes in peripheral blood, comprising 10%–30% of osteoclast precursors, whose differentiation into mature osteoclasts is regulated by the RANKL/OPG balance [44]. Previous studies have shown that elevated inflammatory levels in gout patients lead to suppressed OPG expression due to pro‐inflammatory cytokines (IL‐1β and TNF‐α), thereby promoting osteoclast differentiation and bone resorption [45, 46]. RNA sequencing in this study showed LPS‐induced activation of osteoclastogenic pathways in PBMCs, with in vitro experiments confirming enhanced osteoclast‐specific gene expression and differentiation. Similarly, Roberta Scianaro et al. demonstrated that LPS exposure in osteomyelitis patients' PBMCs increases intracellular inflammation and osteoclast differentiation capacity [47]. Most notably, a significant number of genes related to cellular metabolism exhibited downregulated expression in the RNA‐seq, implicating altered metabolic flux in the process of inflammatory osteoclast differentiation. Therefore, elucidating the mechanisms underlying metabolic reprogramming during inflammatory osteoclast differentiation is crucial for understanding the pathogenesis of inflammatory bone loss and identifying potential therapeutic interventions.

Inflammation‐driven activation of the UPR in the ER is a primary contributor to cellular metabolic and functional alterations. The IRE1‐XBP1s pathway, as one of the three principal UPR branches and the most evolutionarily conserved, plays a pivotal role in metabolic diseases and inflammation [12, 48, 49]. Therefore, it is necessary to further investigate how the IRE1‐XBP1s pathway regulates inflammatory osteoclast formation by modulating cellular metabolism. In the present study, we demonstrated that the IRE1‐XBP1s pathway serves as a central regulator of inflammatory osteoclast differentiation through single‐cell RNA sequencing. Specifically, it showed elevated proportions of XBP1s‐positive osteoclast precursors and osteoclast precursors co‐expressing XBP1s and RANK in LPS‐treated mice. Further in vitro molecular biology experiments demonstrated a positive correlation between the activation/silencing of the IRE1‐XBP1s pathway and the expression levels of osteoclast‐specific genes. In vivo studies revealed that blocking this pathway significantly alleviated bone loss. This observation aligns with previous reports linking UPR to osteoclastogenesis in ovariectomy‐induced bone loss models [16, 50].

Interestingly, further RNA‐seq suggested that the IRE1‐XBP1s regulates osteoclast differentiation by modulating Slc6a4 expression. The Slc6a4 gene encodes the 5‐HT transporter (5‐HTT), which is primarily localized on neuronal cell surfaces and functions to reuptake 5‐HT. This process regulates synaptic 5‐HT concentrations and neural signal transmission. Genetic polymorphisms and aberrant expression of this gene are associated with various disorders, including depression and anxiety [51, 52, 53]. Meanwhile, previous studies have demonstrated that 5‐HTT mRNA is present in osteoclasts, osteoblasts, and osteocytes [54, 55, 56]. The subsequent experiments have confirmed that the translated protein functions to regulate bone cells [47, 54, 56]. In this work, we first confirmed through ChIP‐PCR and dual‐luciferase reporter assays that XBP1s, as a transcription factor, binds to the promoter region of Slc6a4 and exerts regulatory effects in osteoclasts. Moreover, alterations in the activity state of IRE1‐XBP1s consistently correlated with changes in the transcriptional and translational levels of Slc6a4 in vitro, further validating the pivotal role of the IRE1‐XBP1s‐Slc6a4 pathway in inflammatory osteoclastogenesis. Notably, as indicated by the database analyses in Figure 4F, the IRE1‐XBP1s pathway may also exert transcriptional regulation of Slc6a4 in myeloid cells such as dendritic cells, although the downstream regulatory outcomes are likely to be context‐dependent. In parallel, the functional roles of Slc6a4 vary substantially across macrophages, dendritic cells, and neuronal cells, where it is implicated in processes such as inflammatory regulation, antigen presentation, and neurotransmission, respectively. So, the regulatory mechanism of the IRE1‐XBP1s‐Slc6a4 pathway may have potential generalizability in other cell types. Given the emerging evidence indicating that bone tissue itself possesses the capacity for 5‐HT synthesis [56], our ELISA results demonstrated that blocking the IRE1‐XBP1s pathway with 4μ8C led to increased extracellular 5‐HT levels compared to the control group, while intracellular 5‐HT levels decreased correspondingly. These findings provide strong evidence supporting a role for 5‐HT metabolism in osteoclast differentiation. To our knowledge, this regulatory mechanism has not been reported previously.

Notably, central 5‐HT cannot cross the blood‐brain barrier, so only peripheral 5‐HT can directly regulate osteoclastogenesis. Approximately 5% of circulating 5‐HT is stored in platelets and can be released into the bloodstream during systemic inflammatory responses, thereby exerting regulatory effects in peripheral tissues [19, 57]. In order to further elucidate the mechanism by which peripheral 5‐HT metabolism regulates osteoclastogenesis, we conducted both in vivo and in vitro studies, and the results demonstrated that 5‐HT promotes LPS‐mediated osteoclast differentiation, which was consistent with the previous viewpoints [23, 58]. In contrast, we propose that intracellular 5‐HT accumulation exacerbates osteoclastogenesis independently of binding the membrane surface 5‐HT receptors. Because TRAP staining showed that escitalopram (5‐HTT inhibitor) reduces differentiation, while asenapine (5‐HT receptor inhibitor) shows no effect. And the further mice experiments confirmed that escitalopram significantly attenuated the osteoclastogenesis‐promoting effect of 5‐HT in vivo. However, the involvement of other cell types and specific 5‐HT receptor subtypes cannot be entirely excluded and warrants further investigation. Our data suggest that SSRIs may have protective effects against inflammatory bone resorption, a hypothesis that warrants further clinical investigation, particularly in patients with comorbid depression and inflammatory bone diseases. These findings undoubtedly provide new insights into the regulatory role of 5‐HT and 5‐HT transporter inhibitors in bone metabolism.

Since 5‐HT can promote osteoclast differentiation independently of 5‐HT receptors under inflammatory conditions, the underlying mechanism urgently requires further investigation. RNA‐seq analysis revealed that 5‐HT enhances the transcriptional activity of TNFα and IL‐1β in LPS osteoclasts, both of which are well‐established pro‐inflammatory factors that directly promote osteoclast differentiation, a finding supported by our study. Given that RNA‐sequencing of human PBMCs showed significant downregulation of metabolic pathway‐related genes during LPS‐induced osteoclast differentiation, our further untargeted metabolomic analysis demonstrated that 5‐HT reduces the metabolic levels of hundreds of substances in osteoclast precursors with the presence of LPS indeed. Among these altered metabolites, 3MA is of particular interest, as it potently inhibits autophagy which is a process critically implicated in osteoclast differentiation activity. However, in previous studies investigating 3MA‐mediated autophagy inhibition and RANKL‐induced osteoclast differentiation, 3MA was typically applied exogenously without addressing whether cells could synthesize this compound endogenously [59, 60]. To exclude potential false positives in the untargeted metabolomic analysis, we aimed to confirm the presence of 3MA in LPS‐mediated osteoclast and quantitatively assess its metabolic changes under 5‐HT intervention using HPLC‐MS. Importantly, 3MA was consistently detected in all cell samples under identical HPLC‐MS conditions, and intracellular 3MA levels were significantly lower in the 5‐HT‐treated group than in the control group. Therefore, we propose that 5‐HT promotes inflammatory osteoclast differentiation by inhibiting intracellular 3MA synthesis and enhancing cellular autophagy. Although its precise origin remains to be defined, it may arise from inflammation‐associated alterations in purine metabolism or aberrant methylation processes under cellular stress conditions. The previous study suggested that 3MA is not exclusively an exogenous synthetic compound, but can also arise endogenously through DNA alkylation and cellular methylation reactions [61]. From a functional perspective, the accumulation of endogenous 3MA may have physiological relevance by contributing to the suppression of autophagic activity in osteoclasts, thereby influencing their differentiation and function during inflammatory bone resorption. These findings show that 3MA may represent a previously unrecognized bioactive metabolite linking metabolic reprogramming to autophagy regulation in inflammatory settings. These findings also suggest the existence of a unique autophagy regulatory system during inflammatory osteoclast differentiation, providing novel theoretical foundations and research strategies for autophagy‐related studies.

**FIGURE 8:**
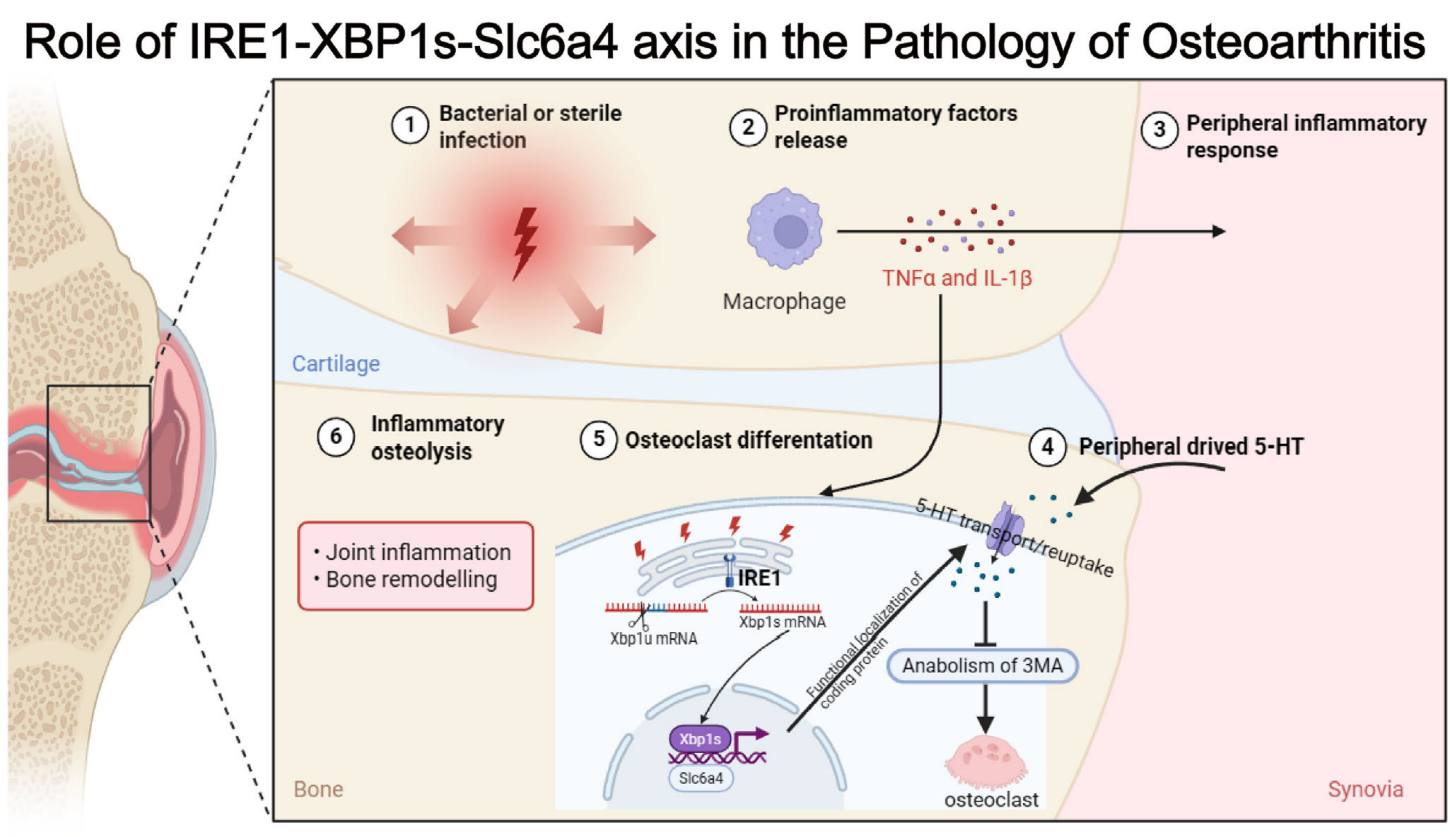
Schematic diagram of the molecular mechanism of inflammatory osteolysis. A graphical abstract for the mechanisms by which IRE1‐XBP1s‐Slc6a4 axis regulates inflammatory osteoclast differentiation through a 5‐HT dependent endogenous anti‐autophagy. The bacterial or sterile inflammation promotes macrophages releasing proinflammatory factors, which can trigger the UPR in osteoclast precursor cells and active the peripheral inflammatory response. IRE1 converts XBP1u to XBP1s by unconventional splicing, which can translocalize to the nucleus and bind to the Slc6a4 promoter to regulate its expression. The 5‐HT transporter, encoded by Slc6a4, colonized the cell membrane and reuptakes the peripheral‐derived 5‐HT driven by inflammation into the cell. By inhibiting 3MA anabolism, 5‐HT can weaken the inherent anti‐autophagy ability of cells and then promote osteoclast differentiation.

The regulatory role of autophagy in OVX‐mediated osteoclast differentiation has been extensively studied [62, 63], and its involvement in periprosthetic osteolysis has been sporadically reported [59, 64, 65]. However, the molecular mechanisms underlying its regulation in LPS‐induced inflammatory osteoclastogenesis remain poorly understood. Subsequent in vitro and in vivo experiments demonstrated that supplementation with 3MA not only significantly inhibited LPS‐mediated osteoclast differentiation but also markedly attenuated the pro‐osteoclastogenic effects of 5‐HT. Previous studies have implicated p62 and TSC1 in the regulation of osteoclast autophagy and differentiation; whether these autophagy‐related regulators participate in the IRE1‐XBP1s‐Slc6a4‐5‐HT axis identified in the present study remains to be determined [66, 67, 68]. ELISA analysis confirmed that 3MA exerts its inhibitory effects on inflammatory bone resorption by suppressing the synthesis and secretion of TNF‐α. To summarize, we conclude that LPS‐mediated inflammatory stimulation induces UPR in osteoclast precursor cells, activating the IRE1‐XBP1s pathway. This subsequently enhances 5‐HT transporter function and increases 5‐HT reuptake, further promoting inflammatory osteoclast differentiation. Mechanistically, 5‐HT inhibits intracellular 3MA synthesis, thereby promoting pro‐inflammatory cytokine production and autophagy during inflammatory osteoclastogenesis (Figure 8). HPLC‐MS analysis revealed that escitalopram suppresses LPS‐induced osteoclastogenesis by upregulating 3MA synthesis and inhibiting autophagy. Our experimental findings provide novel insights into the therapeutic potential of these drugs. Current clinical management of inflammatory bone resorption remains suboptimal, and while combined anti‐inflammatory and anti‐osteoclastogenic therapy is theoretically promising, it lacks robust mechanistic support. This study reveals that osteomyelitis patients exhibit not only increased number of osteoclasts at lesion sites but also significantly enhanced osteoclastogenic potential in PBMCs. Notably, pharmacological blockade of the IRE1‐XBP1s pathway and inhibition of 5‐HT transporter activity both effectively suppress this pathological process. Furthermore, similar pharmacological effects were observed in PBMCs from RA and OA patients, suggesting a conserved mechanism across inflammatory bone disorders. These findings suggest that SSRIs may emerge as a novel class of therapeutic agents for inflammatory bone diseases.

While our study provides compelling evidence for the role of the IRE1‐XBP1s‐Slc6a4 pathway in inflammatory osteoclastogenesis, several limitations should be acknowledged. First, the primary focus on LPS‐mediated inflammation may not fully capture the complexity of polymicrobial infections or sterile inflammatory conditions. In addition, although our in vitro time‐course experiments demonstrated early activation of the IRE1‐XBP1s pathway following LPS stimulation, early in vivo signaling kinetics were not evaluated in the current study. Future studies should explore the pathway's involvement in other inflammatory models, such as those driven by Staphylococcus aureus or autoimmune triggers, and further characterize the temporal dynamics of IRE1‐XBP1s activation in vivo. Second, the mechanistic link between 5‐HT transport and 3MA metabolism remains incompletely understood. Further research is needed to elucidate the enzymatic pathways involved in 3MA synthesis and degradation in osteoclasts, as well as the precise molecular mechanisms by which 5‐HT modulates these processes. Third, while our in vivo data support the therapeutic potential of targeting the IRE1‐XBP1s‐Slc6a4 axis, the long‐term pharmacological safety and efficacy in humans require rigorous clinical evaluation. Preclinical studies in large animal models and clinical trials will be essential to advance these findings toward clinical application.

## 5 Conclusion

In summary, this study establishes the IRE1‐XBP1s‐Slc6a4 signaling axis as a critical regulator of inflammatory osteoclastogenesis and osteolysis. By linking ER stress, 5‐HT metabolism, and autophagy modulation, our findings provide a unified mechanistic framework for understanding inflammatory bone loss and offer novel therapeutic strategies for orthopedic infections and inflammatory bone diseases. The translational potential of targeting this pathway, particularly with repurposed drugs like escitalopram, underscores the importance of further research in this area. Ultimately, these insights may lead to more effective treatments for patients suffering from debilitating bone resorption disorders.

## Author Contributions

**Pengchao Yang**: methodology, software, writing – original draft, investigation, data curation, formal analysis, visualization. **Binxiang Zhu**: validation, formal analysis, visualization. **Yuzhi He**: investigation, software, formal analysis, validation. **Yang Tian**: methodology, software. **Honglei Kang**: methodology, conceptualization, funding acquisition. **Shian Hu**: investigation, software. **Pengju Wang**: software, formal analysis. **Yong Xu**: methodology, data curation. **Zhuowei Lei**: methodology. **Peijun Qi**: methodology. **Hao Yang**: methodology. **Yang Lin**: methodology. **Yimin Dong**: conceptualization, methodology, funding acquisition, writing – review and editing, resources. **Feng Li**: conceptualization, project administration, funding acquisition, supervision, writing – review and editing, resources. **Hanfeng Guan**: conceptualization, writing – review and editing, project administration, resources, supervision.

## Conflicts of Interest

The authors declare no conflicts of interest.

## Supporting information




**Supporting File 1**: advs76755‐sup‐0001‐FigureS1‐S7.docx.

**Supporting File 2**: advs76755‐sup‐0001‐TableS1.docx.

## Data Availability

Other data supporting the findings of this study are available from the corresponding author upon reasonable request. The single‐cell RNA sequencing data generated in this study have been deposited in the OMIX database of the China National Center for Bioinformation under accession number OMIX018263, and the untargeted metabolomics data have been deposited under accession number OMIX018260. These datasets will be publicly accessible upon publication.
