# Supplementary material for: The IRE1‐XBP1s Axis Drives Inflammatory Osteolysis by Regulating a 5‐HT Dependent Endogenous Anti‐Autophagy Mechanism
Source: Adv Sci (Weinh). 2026 Jul 23;13(58):e76755. doi: 10.1002/advs.76755 (PMC13395397; doi:10.1002/advs.76755)
Supplement: Supplementary file 1 [file ADVS-13-e76755-s001.docx]

Supplementary Table 1. The basic clinical information of healthy donors and patients.

| Name | ID | Gender | Age (y) | Diagnosis |
| --- | --- | --- | --- | --- |
| Zhiming Wang | 60017996593 | Male | 31 | healthy donor |
| Yanhua Deng | 60017813347 | Female | 69 | healthy donor |
| Zusong Xiao | 60018146672 | Male | 37 | healthy donor |
| Zhengmei Liu | 60018514139 | Female | 55 | healthy donor |
| Yanjun Liu | 60018448713 | Female | 57 | healthy donor |
| Zhaoang Xu | 60017817770 | Male | 58 | healthy donor |
| Wencan Qian | 60016945994 | Male | 31 | osteomylitis |
| Xiaogang Guo | 60012490443 | Male | 56 | osteomylitis |
| Boming Zhang | 60016798168 | Male | 70 | osteomylitis |
| Jiaqiong Li | 60014665387 | Female | 34 | osteomylitis |
| Lamei Wang | 60017664529 | Female | 62 | osteomylitis |
| Jinhua Tu | 60018316182 | Female | 46 | osteomylitis |
| Dongsheng Li | 60017801227 | Female | 62 | rheumatoid arthritis |
| Changfeng Cao | 60017025208 | Female | 57 | rheumatoid arthritis |
| Yajuan Xiang | 60010158127 | Female | 47 | rheumatoid arthritis |
| Jinfeng Yuan | 2501972258 | Female | 54 | rheumatoid arthritis |
| Fenglian Cao | 60015907251 | Female | 39 | rheumatoid arthritis |
| Xiaohong Rong | 60016021824 | Female | 55 | rheumatoid arthritis |
| JingE Deng | 60010922410 | Female | 76 | osteoarthritis |
| Daoqin Qian | 60015745291 | Female | 62 | osteoarthritis |
| Liping Sun | 60016623185 | Female | 60 | osteoarthritis |
| Hehua Chen | 60018802587 | Female | 56 | osteoarthritis |
| Huaiju Sheng | 60016566137 | Female | 59 | osteoarthritis |
| Huosheng Gu | 60016116397 | Male | 65 | osteoarthritis |
