# Supplementary material for: The IRE1‐XBP1s Axis Drives Inflammatory Osteolysis by Regulating a 5‐HT Dependent Endogenous Anti‐Autophagy Mechanism
Source: Adv Sci (Weinh). 2026 Jul 23:e76755. Online ahead of print. doi: 10.1002/advs.76755 (PMC13395397; doi:10.1002/advs.76755)
Supplement: Supplementary file 1 — Supporting File 1: advs76755‐sup‐0001‐FigureS1‐S7.docx. [file ADVS-9999-e76755-s001.docx]

Figure S1.

Figure S2.

Figure S3.

Figure S4.

Figure S5.

Figure S6.

Figure S7.

Figure legend:

Figure S1:

(A) Schematic representation of LPS-mediated inflammatory osteoclast differentiation.

(B-C) Representative TRAP staining images (B) and quantification (C) of mature osteoclasts with LPS stimulation. Scale bars: 200 μm.

(D) mRNA expression of osteoclast-associated genes in BMDMs with LPS stimulation.

(E-F) Representative fluorescence images (E) and quantification (F) of actin rings with LPS stimulation. Scale bars: 200 μm.

(G) qPCR result shows the expression of IL-1β and TNFα with the stimulation of LPS.

(H-I) The protein expression and quantitative analysis of IRE1, p-IRE1 and XBP1s with LPS intervention at different time point.

(J) ‌Schematic diagram of LPS intervention method during the modeling of inflammatory osteolysis.

Figure S2:

(A) mRNA expression of XBP1s in BMDMs with OE-XBP1s adenovirus transfection.
(B) Quantification of mature osteoclasts with OE-XBP1s adenovirus transfection.
(C) The quantitative analysis of protein in BMDMs with OE-XBP1s adenovirus transfection.

(D-G) The protein expression and quantitative analysis of IRE1, p-IRE1 and XBP1s with the treatment of TG.

(H-I) Representative TRAP staining images (H) and quantification (I) of mature osteoclasts with TG stimulation. Scale bars: 200 μm.

(J) qPCR result shows the expression of osteoclast-special genes with the stimulation of TG.

(K) mRNA expression of IRE1 in BMDMs with sh-IRE1 adenovirus transfection.

(L) Quantification of mature osteoclasts with sh-IRE1 adenovirus transfection.

(M) The quantitative analysis of protein in BMDMs with sh-IRE1 adenovirus transfection.

(N) The cell viability analysis of BMDMs exposed to different dose 4μ8C.

(O-P) The 4μ8C treatment can alleviate the osteoclast differentiation-promoting effects of IL-1β and TNFα. Scale bars: 200 μm.

Figure S3:

(A) Representative toluidine blue staining images of calvarial slices from each group. Black arrows indicate osteoclasts.

(B-C) Quantification of osteoblast-related histomorphometric parameters N.OB/B.Pm and OB.S/B.S (n≥6).

(D) The concentration of PINP in the serum of each group of mice.

Figure S4:

(A) mRNA expression of Slc6a4 in BMDMs with OE-Slc6a4 adenovirus transfection.
(B) qPCR result shows the expression of osteoclast-special genes with the transfection of OE-Slc6a4.

(C-D) Representative TRAP staining images (C) and quantification (D) of mature osteoclasts with 5-HT stimulation. Scale bars: 200 μm.

(E) Quantification of mature osteoclasts with escitalopram stimulation.

(F) Quantification of mature osteoclasts with asenapine stimulation.

(G) qPCR result shows the expression of osteoclast-special genes with the stimulation of escitalopram.

(H-I) The cell viability analysis of BMDMs exposed to different dose escitalopram (H) and asenapine (I).

(J) H&E staining of the heart, liver, and kidney tissue in each group. Scale bars, 200 μm.

(K) Representative toluidine blue staining images of calvarial slices from each group. Black arrows indicate osteoclasts.

(L-M) Quantification of osteoblast-related histomorphometric parameters N.OB/B.Pm and OB.S/B.S (n=6).

Figure S5:

(A) Quantification of mature osteoclasts with TNFα stimulation.

(B) Quantification of mature osteoclasts with IL-1β stimulation.

Figure S6:

(A) The cell viability analysis of BMDMs exposed to different dose 3MA.

(B-C) Representative TRAP staining images (B) and quantification (C) of mature osteoclasts with supplement of 3MA. Scale bars: 200 μm.

(D) qPCR shows the mRNA levels of autophagy associative genes with supplement of 3MA.

(E) The protein quantitative analysis of autophagy associative genes with supplement of 3MA.

(F-G) Representative TRAP staining images (F) and quantification (G) of mature osteoclasts with TG and 3MA stimulation. Scale bars: 200 μm.

(H) H&E staining of the heart, liver, and kidney tissue in each group. Scale bars, 200 μm.

(I) Representative toluidine blue staining images of calvarial slices from each group. Black arrows indicate osteoclasts.

(J-K) Quantification of osteoblast-related histomorphometric parameters N.OB/B.Pm and OB.S/B.S (n=6).

(L-N) Quantification of human mature osteoclasts induced from PBMCs of osteomylitis (L), RA (M), and OA (N) patients compared to healthy donors with the stimulation of 4μ8C and escitalopram.

Figure S7:

Full-length, unedited immunoblots corresponding to the Western blot figures presented in the main manuscript. The cropped blots shown in the figures were derived from these original images.
